# Atmospheric Mixing Controls Air Pollution Chemistry and Mitigation Efficacy in High-Latitude Cities

**DOI:** 10.1021/acs.est.6c00181

**Published:** 2026-07-23

**Authors:** Jonas Kuhn, Laura M. D. Heinlein, Meeta Cesler-Maloney, Michael O. Sunday, Fangzhou Guo, James H. Flynn, Allison Moon, Becky Alexander, William R. Simpson, Cort Anastasio, Jochen Stutz

**Affiliations:** † Department of Atmospheric and Oceanic Sciences, 8783 University of California, Los Angeles, California 90095, United States; ‡ Department of Land, Air, and Water Resources, 8789 University of California, Davis, California 95616, United States; § Department of Chemistry and Biochemistry, 11414 University of Alaska, Fairbanks, Alaska 99775, United States; ∥ Department of Earth and Atmospheric Sciences, 14743 University of Houston, Houston, Texas 77204, United States; ⊥ 53777 Aerodyne Research Inc., Billerica, Massachusetts 01821, United States; # Department of Atmospheric and Climate Science, 7284 University of Washington, Seattle, Washington 98195, United States

**Keywords:** air pollution, arctic, atmospheric oxidation, secondary aerosol, mitigation

## Abstract

Urban air pollution in cold environments poses a significant public health risk. However, the physicochemical processes determining the concentrations of primary and secondary pollutants remain poorly understood. This is due to fundamentally different conditions compared to warmer environments, such as extremely shallow polluted surface layers (PSLs) and low ultraviolet radiation. We apply an observation-driven chemical transport model to a multiday persistent PSL event during the 2022 Alaskan Layered Pollution and Chemical Analysis (ALPACA) experiment in Fairbanks, AK, USA. The simulations account for pollutant emissions, multiphase chemical kinetics, and turbulent and advective exchange of PSL air with the clean background atmosphere. This exchange is continuous, occurs on a time scale of 30 min to 3.5 h, and is essential for an accurate representation of the PSL composition. We find that measured diurnal cycles of particulate nitrate reflect the interplay between photochemical nitric acid formation and the loss of nitrate aerosol from of the PSL through mixing with clean background air. The continuous removal and replenishment of PSL aerosol counteracts self-acidification and prevents coagulation with acidic primary sulfate aerosol, thus sustaining sulfate and hydromethanesulfonate (HMS) formation throughout the pollution event. Sensitivity calculations show that within the coupled chemistry and transport system of a shallow PSL, reductions of nitrogen oxide emissions can change the multiphase oxidation regime and thereby even increase the levels of secondary nitrate and sulfate in the PSL.

## Graphical abstract

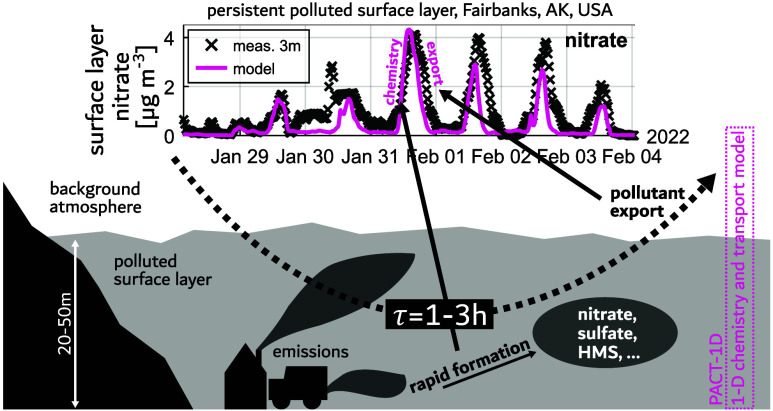

## Introduction

Frequent extreme air pollution episodes in cold-climate urban environments represent a serious public health hazard. During winter, weak surface winds, low insolation, and high thermal emissivity of snow cause the formation of very shallow surface layers through strong inversions in the near-surface temperature profile. The reduced vertical mixing with the background atmosphere leads to the accumulation of pollutants from different sources, such as residential heating or traffic, near the surface.^28,31^ These shallow layers can persist for days, dramatically enhancing exposure of the urban community to high concentrations of particulate matter (PM) and gaseous pollutants, such as sulfur dioxide (SO_2_) and nitrogen oxides (NO_X_), with a severe negative impact on human health.^18^

The composition and chemistry of cold and shallow polluted surface layers (PSLs) is fundamentally different from the well-studied air pollution scenarios in lower-latitude environments: (a) Extraordinarily high concentrations of trapped pollutants and region specific pollutant sources contribute to a unique composition of the polluted air mass. For instance, home heating with wood burning and sulfur-rich oil is a dominant emission source. (b) This air mass occupies a relatively small volume as it is confined to only a few dozen meters above the surface,^25,26^ enhancing the influences of residual mixing with the cleaner background air aloft. Furthermore, (c) temperatures and ultraviolet (UV) actinic fluxes are strongly reduced, leading to a unique chemical regime.

The oxidation of emitted gases forms secondary pollutants, e.g., SO_2_ and NO_X_ oxidation forming sulfate and nitrate, contributing to fine PM. At lower latitudes, atmospheric oxidation is governed by OH radical formation initiated by UV photolysis of ozone.^9,19^ In contrast, during high-latitude winters, production of OH and its reaction products HO_2_ and H_2_O_2_ is expected to be drastically reduced due to low UV actinic fluxes, implying low rates of secondary pollutant production in the gas and particle phase. However, PSLs contain high amounts of sulfate and nitrate^7,25^ with an essential contribution of secondary production.^14,22^ This points to the relevance of other oxidant sources, including the photolysis of nitrous acid (HONO) or formaldehyde (HCHO), the reaction of ozone with alkenes,^13^ or the formation of NO_3_ radicals from ozone and NO_2._ ^5^ Moreover, in-particle oxidant formation from the photochemistry of light-absorbing organic carbon (i.e., brown carbon, BrC) has been proposed.^14,27^ Other particle-phase oxidation reactions, e.g., with dissolved NO_2_ or peroxynitric acid (PNA) can also play an important role.^30,32^ In general, chemical processing in the particle phase is favored since cold temperatures drive semivolatile species toward the particle phase. This is also important for the explanation of the high amounts of hydroxymethanesulfonate (HMS), which have been observed in the PSL.^6^ HMS is formed by the reaction of HCHO with sulfite (SO_3_^2–^) in the particle phase and, thus, depends on high solubility of both HCHO and ammonia, which controls particle acidity.^2,7^ The relative importance of the different chemical conversion pathways for secondary pollutant formation in the PSL air mass remain unclear, mostly due to poorly quantified oxidant amounts and large uncertainties in particle composition, mixing state, and acidity.

A major challenge in understanding and quantifying the PSL chemical system is the role of mixing with the clean background atmosphere. The PSL can be regarded as a transient chemical reactor in which high concentrations of pollutants and high chemical conversion rates are sustained by continuous surface emissions of pollutants and their gradual removal from the PSL. The removal of pollutants proceeds mostly by advection, which is most efficient when the polluted air mass vertically mixes into the background air above the PSL, where wind speeds are higher. Deposition plays a minor role (ca. 1–10%, see Figure S1). The exchange with the background atmosphere will (a) limit the residence time of pollutants within the PSL, constraining chemical processing time and eventually determining the pollutant concentrations, and (b) drive the entrainment of clean background air into the PSL. Under heavily polluted conditions, O_3_ levels are often depleted by the reaction with NO.^1,24,25^ This is a dynamic process, where O_3_ continuously mixed in from the background atmosphere reacts rapidly with abundantly emitted NO.^26^ Consequently, the time scale of mixing with background air determines the response of the PSL chemical system to changing pollutant emission rates and background oxidant levels. In addition, the pollutant residence time in the PSL limits the time for particles from different sources to mix and coagulate, and thus controls the mixing state of the aerosol. Despite its critical influence on PSL chemistry, the exchange of the polluted air with the clean oxidant-rich background remains poorly studied and is often ignored in the interpretation of measurements.

This work aims to improve our understanding of the surface layer composition in cold, polluted environments by quantifying the exchange of the PSL with background air, investigating oxidant formation mechanisms, and analyzing the relative importance of the oxidative and nonoxidative pathways leading to secondary aerosol formation. Specifically, we aim to answer the following questions: Which processes are responsible for the high amounts of secondary inorganic aerosol in cold and shallow PSLs? How does atmospheric mixing influence secondary aerosol formation and measured secondary aerosol levels? How does the PSL chemical system react to different emission reduction scenarios? We focus on the formation of the high nitrate, secondary sulfate, and HMS levels observed in a cold, polluted episode during the “Alaskan Layered Pollution and Chemical Analysis” (ALPACA) field campaign in Fairbanks, AK, USA during the winter of 2022.^25^ Observation-driven modeling of multiphase chemical kinetics in the polluted air alongside emission, vertical dispersion and advective export from the PSL can reproduce the observed secondary pollutant amounts. It highlights the complexity of the unique chemical PSL system, which is heavily influenced by the exchange with clean background air.

## Methods

### Observation-Driven Modeling of the Composition and Chemistry in a Cold and Shallow PSL in Fairbanks, AK, USA

We study the composition and chemistry in cold and shallow PSLs using an observation-driven modeling approach. We combine our knowledge of (1) the atmospheric state, from observations, (2) pollutant emission rates, from inventories, and (3) chemistry, from literature mechanisms, in a model to simulate the PSL composition. The comparison between the simulated and the measured PSL composition then allows us to understand the underlying chemical and transport processes. This integrated approach provides a general framework for quantifying how the coupling of multiphase chemistry and atmospheric mixing controls pollutant levels in localized air masses and allows the investigation of potential mitigation strategies.

Observation-driven modeling requires comprehensive observational data. This study relies on data acquired during the intensive ALPACA field campaign, conducted in Fairbanks, AK, USA in Jan. and Feb. 2022.^25^ Fairbanks is located in a basin where meteorological conditions during winter lead to the frequent formation of PSLs. Here, we focus on a sustained cold (243–255 K) and heavily polluted PSL event between 29 Jan. and 4 Feb. 2022, which is typical for midwinter Fairbanks and provides ideal conditions for our case study, i.e., shallow PSL heights and high pollutant concentrations.^8^

In order to provide mechanistic understanding of the multiphase composition of the PSL air mass, we expanded and adapted the one-dimensional chemistry and transport model “Platform for Atmospheric Chemistry and Transport in one dimension” (PACT-1D).^17,26,29^ Reducing the spatial heterogeneity to a single vertical dimension enables the detailed treatment and tracing of explicit multiphase chemical kinetics, which is needed to disentangle different pollutant formation pathways under the influence of mixing. PACT-1D accounts for gas emissions, deposition, vertical mixing, advective exchange with background air and chemistry averaged within a 4 by 4 km area covering downtown Fairbanks (Figure 1A). The atmosphere is divided into a vertical stack of 39 homogeneously mixed boxes. The size of the boxes was chosen to match the spatiotemporal dimension of the processes under study, i.e., 10 m above 80 m, 3–5 m below 80 m, and logarithmically decreasing within the last meter above ground.^17^

**Figure 1:**
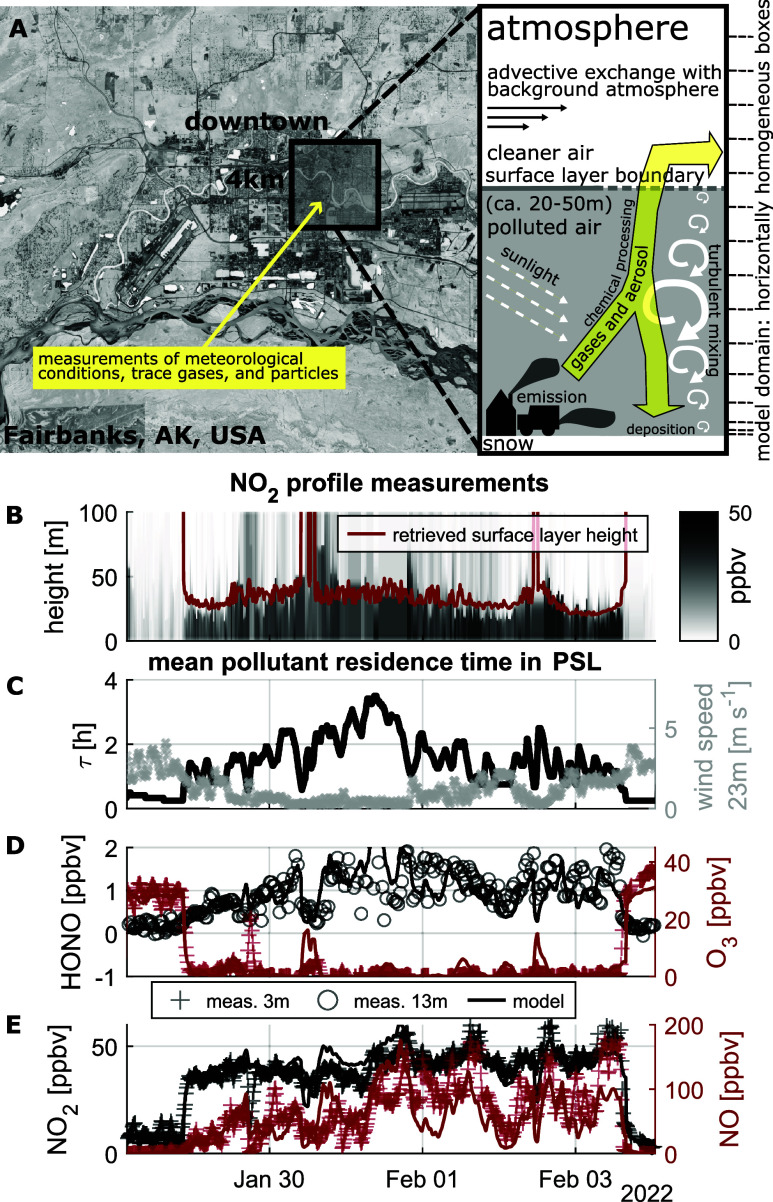
Satellite image (2026 Google) of Fairbanks, AK, USA with black square marking the 4 km by 4 km model area containing the downtown area (A). Pollutants are emitted into the horizontally homogeneous model domain, which can mix with clean background air. The surface layer height has been inferred from trace gas profile measurements (B) and parametrizes the turbulent diffusivity profiles in the model. After their emission, pollutants reside only a few hours in the surface layer, before being mixed upward and removed by advection at the surface layer top (C). The turbulence-advection scheme, together with gas-phase chemistry, accurately reproduces measured HO_X_ precursors (D), NO_X_ (E), and other gaseous pollutants (Figures S2 and S3).

Ambient conditions measured during ALPACA serve as static boundary conditions, *b*, constraining the evolution of the atmospheric state within the model domain. These boundary conditions include measurements, such as temperature, pressure, wind speed, and photolysis frequencies, derived quantities, such as molecular diffusivities, chemical reaction rates, and uptake coefficients, and surface pollutant emissions from inventories. The state vector *y* contains the vertical distribution of the concentrations of trace gases and dissolved species. The differential equation

$$\frac{dy}{dt}=f\left(y,b\left(t\right)\right)=f_{\mathrm{ch}}\left(y,b\left(t\right)\right)+f_{tr}\left(y,b\left(t\right)\right)$$

is integrated by consecutively solving the transport *f*_tr_ and chemistry part *f*_ch_ of the equation.^29^ Sensitivity studies showed that a time step of 5 min for switching between the two solvers yields consistent results in this environment. The transport part combines vertical turbulent diffusion *V*, exchange with the background atmosphere through horizontal advection *H*, dry deposition *D*, and emissions *E*

1 $$f_{tr}\left(y,b\left(t\right)\right)=E\left(b\left(t\right)\right)+V\left(y,b\left(t\right)\right)+H\left(y,b\left(t\right)\right)+D\left(y,b\left(t\right)\right)$$

The vertical diffusion scheme *V* uses an empirical formula for vertical turbulent diffusivity (*K*_*z*_) profiles of Brost and Wyngaard^4^

$$K_{z}\left(z\right)=\kappa u{\;}^{*}h_{S}\frac{(z/h_{S}){\left(1-z/h_{S}\right)}^{1.5}}{1+4.7\left(z/h_{S}\right)\left(h_{S}/L\right)}$$

with height above ground *z*, surface layer heigth *h*_S_, von Karman constant κ = 0.4, a friction velocity of u* = 0.45 ms^–1^ and the Monin-Obukov length *L* assumed to be on the order of *h*_S_(*h*_S_/*L* = 1.4). Thereby we are able to approximate realistic profiles of *K_z_* based on surface layer heights retrieved from trace gas vertical profile measurements (see Figure 1).^26^ When no PSL is present, we use a constant *K_z_* profile with 10 m^2^ s^–1^ .

Exchange with clean background air [35 ppbv O_3_, negligible pollutant concentrations, see ref 25] through horizontal advection *H* is implemented using exchange coefficients *k*_u_ = *u*/*L*_F_, with the wind speed *u* and the horizontal scale of the model domain *L*_F_ = 4 km.

We use measurement data recorded in Fairbanks during ALPACA, which have been published and described in detail elsewhere [refs 7,16,25,26 and Supporting Information]. Emission rates of gaseous pollutants were compiled by USEPA [with modified NO_X_, see ref 3, and Supporting Information]. Aqueous-phase particles are emitted to our model domain in amounts and sizes reflecting measured aerosol liquid water (ALW) and PM1 [see refs 7,16 and Figures S1 and S4]. The particles are considered spherical with 150 nm radius^23^ and undergo the same turbulent and advective transport as the gases. In order to approximate temporally resolved particle emission rates, we scaled the EPA reported methane emission rates, which yields good agreement between measured and modeled ALW and PM1 (Figures S1 and S4). Deposition of particles and gases in snow was adapted to reproduce the results of Kuhn et al., 2025.^17^

The chemistry part *f*_ch_ uses a fully kinetic treatment of the entire multiphase chemical system, without relying on thermodymic equilibrium assumptions. We use the RACM2 mechanism for gas-phase photochemistry,^12^ kinetic exchange between gas and liquid aerosol phase based on Henry’s law, and liquid-phase chemistry in aerosol particles (see Supporting Information). The aerosol pH is calculated directly from H^+^ concentrations, which are controlled by in-particle reaction rates.

Because the time scales of transport processes and chemistry in the PSL overlap, the integrated simulation of PSL chemistry and transport is essential. For instance, PACT-1D is able to model multiphase chemical conversions within the constraint of physical export of gaseous and particulate pollutants from the PSL (e.g., through advection). To clarify the discussion of our results, we introduce the pollutant residence time in the PSL as the time scale related to transport processes.

### Residence Time of Pollutants in the PSL

For the discussion of PSL chemistry it is helpful to introduce the effective residence time τ of a pollutant within the PSL (Figure 1C). It is defined as the time after which an emitted inert pollutant was removed from the PSL through transport with a probability of 1 – *e* ^–1^ ≈ 62%. In other words, the pollutant residence time is the lifetime of a pollutant inside the PSL with respect to physical transport. Thus, it can be calculated using the transport scheme of our model.^26^ The instantaneous residence time scale τ^*^(*t*) (that is the residence time at time t assuming a stationary setting) can be calculated from the loss rates due transport processes (*H* ^†^, *V* ^†^, *D* ^†^) and the vertically integrated pollutant concentration *c̅* in the surface layer

$$\tau^{*}=\frac{\mathrm{c̅}}{H^{†}+V^{†}+D^{†}}=\frac{\int_{0}^{h_{S}}c\left(z\right)dz}{\int_{0}^{h_{S}}Hdz+\int_{0}^{h_{S}}Vdz+\int_{0}^{h_{S}}Ddz}$$

with *H*, *V*, *D* as introduced in eq 1. However, the instantaneous residence time scale τ^*^ does not necessarily represent the residence time of a particle within the surface layer. For example, if, at a given time *t*, τ^*^ is 3 h but 1 h later changes to 1 h, then the effective pollutant residence time at *t* is smaller than 3 h. Thus, the actual effective pollutant residence time τ at a given time *t* depends on the future development of τ^*^. This is expressed by the integral

$$\int_{t}^{t+\tau \left(t\right)}\frac{1}{\tau^{*}\left(t^{\prime }\right)}dt^{\prime }=1$$

which can be solved numerically to provide the temporal evolution of τ (as shown in Figure 1C). The effective residence time τ provides a fundamental temporal constraint to chemical conversion processes in the PSL.

## Results and Discussion

### The PSL Composition is Governed by Mixing with Background Air

The simulated temporal evolution of gaseous pollutant concentrations are in excellent agreement with observations [see Figures 1D,E, S2, S3, and ref 26]. NO_2_ levels increase rapidly upon surface layer formation, accompanied by fast O_3_ loss. This illustrates the sudden trapping of emitted pollutants and the rapid conversion of NO to NO_2_, depleting O_3_ within the PSL. Within ca. 1 h, NO_X_ concentrations level off at relatively constant values that are maintained throughout the entire pollution event. This reflects a quasi-steady state, which adapts dynamically to changes in NO_X_ emission, O_3_ entrainment and related NO to NO_2_ conversion, and NO_X_ removal from the PSL. These characteristics of PSL gas-phase NO_X_ chemistry point toward the relevance of mixing in PSL chemistry and are discussed in detail in Stutz et al., 2026,^26^ who use the same PACT-1D model setup without multiphase chemistry. Their comparison of measured and modeled trace gas profiles underlines the excellent capability of our PACT-1D setup to model the PSL dynamics.

During the studied 5-day pollution period, the pollutant residence time τ in the PSL ranges from about 30 min up to 3.5 h (Figure 1C), which means that during the sustained PSL event, the entire PSL air mass was exchanged about 80 times. This contradicts the widespread belief that PSLs can be considered stagnant air masses able to host slow chemical conversions. Thus, we confirm our finding with a model-independent back-of-the-envelope calculation: Because less than 7% of the SO_2_ emissions react inside the PSL,^22^ we can use SO_2_ as an inert transport tracer. Assuming a PSL height of 30 m (Figure 1B), the SO_2_ emission rate reported by the EPA during the study period can be converted to 10.8–14.4 ppb h^–1^. The steady state SO_2_ mixing ratio in the PSL is determined by the product of the emission rate and the pollutant residence time. A residence time of 1.5 h, as determined by our model, yields an SO_2_ mixing ratio of 18–22 ppb, matching the observations in the PSL (Figures S1 and S2). If the PSL was stagnant with an assumed pollutant residence time of 15 h, the SO_2_ mixing ratio would steadily increase over 15 h and eventually level off at 180–220 ppb. This contradicts the measurements and, to the best of our knowledge, has never been observed in a cold and shallow PSL.

The same steady state reasoning applies to secondary species: for instance, sustaining an observed 1–4 μg m^–3^ of nitrate at residence times of 1–3.5 h requires a production rate of ca. 0.3–1.5 μg m^–3^ h^–1^. Besides constraining chemical processing, the range of pollutant residence times point to a number of further important properties of the PSL. For instance, it can reveal whether aerosol emitted from different sources has sufficient time to become internally mixed while residing in the PSL. Model studies estimate that in polluted air the time scale for externally mixed aerosol (particles with different composition from different sources coexist) to reach an internally mixed state (all particles have the same composition) through diffusion and coagulation is at least on the order of hours.^10^ This is similar to, or longer than, the residence time of the aerosol in the surface layer. Consequently, we expect that substantial parts of the PSL aerosol phase, which also originates from different, spatially separate sources, remain externally mixed. Single particle measurements confirm this assumption by showing a diverse mixture of particle types with different structure, composition, and source processes.^15^ This presents a significant challenge to the assessment of particle chemistry and thermodynamics based on bulk aerosol measurements and implies possible shortcomings of widely used equilibrium assumptions. This also supports our choice of using a kinetic treatment of multiphase aerosol chemistry to study chemical processes in different particle types (see below).

In the following, we use our model setup to investigate the pathways of secondary inorganic aerosol formation in the cold and shallow PSL.

### PSL Nitrate is Formed by Rapid HO_X_ Chemistry During Daytime

Trace gas measurements during the ALPACA campaign are accurately reproduced by PACT-1D, including the main HO_X_ precursors: O_3_, HONO, and HCHO. O_3_ is depleted in the surface layer near the ground, while HONO levels (up to 2 ppb) and HCHO levels (up to 6 ppb) are high (see Figures 1D and S3). The simulations show that gas-phase HO_X_ production in the PSL is dominated by HONO photolysis $\left(\mathrm{HONO}\overset{\mathrm{hv}}{\to }\mathrm{NO}+\mathrm{OH}\right)$ with minor contributions from HCHO photolysis and the reaction of ozone with alkenes (Figure 2). Volatile organic compounds are considered in the simulations but play a minor role in this high-NO_X_ environment, where, due to high rates of NO + HO_2_ → OH + NO_2_, OH to HO_2_ ratios are on the order of unity.

**Figure 2:**
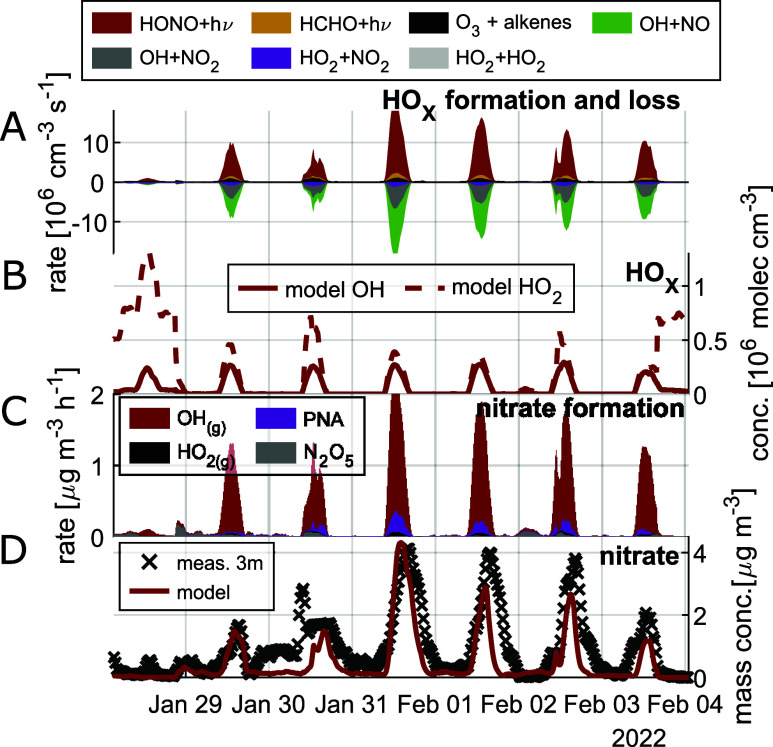
Under polluted conditions, HO_X_ production is determined by HONO photolysis and NO and OH reforming HONO (A). Nitric acid formation through OH + NO_2_ is the major terminal HO_X_ sink. High NO levels lead to OH and HO_2_ ratios close to unity (B), due to the high rate of the NO + HO_2_ → NO_2_ + OH reaction. OH levels reached 3 ·10^–5^ molecules cm^–3^ during daytime. Most particulate nitrate in the PSL originates from the nitric acid produced in the gas phase, partitioning into the aerosol (C). Modeled nitrate concentrations excellently agree with temporally resolved measurements^7^ (D). The diurnal cycle reflects photochemical production and continuous removal of the secondary nitrate aerosol from the PSL.

Almost all HO_X_ is lost through the reactions of OH with NO, reforming HONO and the reaction of OH with NO_2_, producing nitric acid (HNO_3_). The partitioning of this HNO_3_ into the aerosol phase represents the dominant particulate nitrate source (Figure 2C). The diurnal cycle in PSL particulate nitrate measurements with peak nitrate levels during daytime (Figure 2D) is due to the nitric acid aerosol uptake which begins when production starts after sunrise. Our simulations show that the falloff of nitrate levels, starting during sunset to near-zero levels at night, reflects the removal of the nitrate-containing aerosol from the PSL by mixing with the background atmosphere. These results show that the observed high levels of particulate nitrate are formed in the PSL and confirm that surface pollutant levels, during the course of a persistent PSL event, are controlled by continuous exchange between the PSL and the clean background air mass.

### Continuous Pollutant Emissions and Atmospheric Mixing Enable Rapid and Persistent Particulate Sulfur Formation in the PSL

High amounts of particulate sulfate were observed during cold polluted ALPACA periods (exceeding 10 μg m^–3^).^25^ Most of the sulfate was found in small particles (ca. 90% in particles with radius *r* < 0.7 μm).^22^ The isotopic composition of the sulfate molecules indicates substantial primary emissions (>60% of the measured sulfate in all 12–24 h samples).^22^ Mass concentrations of secondary sulfate and HMS (see Figure 3A,B) are similar to the observed nitrate levels; however, because the gas-phase oxidation of SO_2_ is slow, they are predominantly formed in the aerosol phase.^14,22^

**Figure 3:**
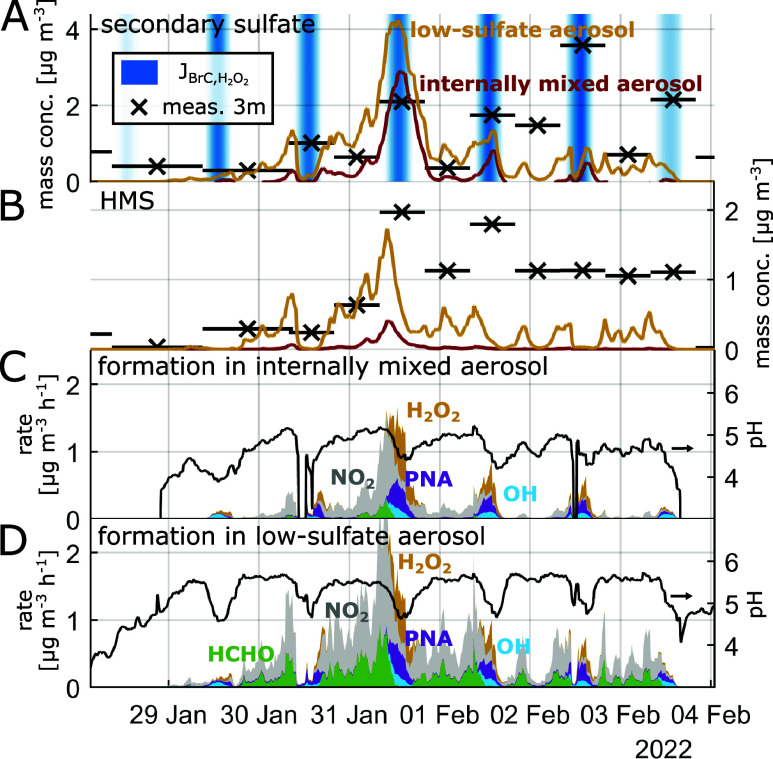
Measured secondary sulfate (A) and HMS (B)^22^ compared with simulated amounts assuming an internally mixed aerosol phase (red) and the low-sulfate component of an externally mixed aerosol (yellow). The blue shaded areas show the photolysis frequencies of BrC to indicate daytime hours. Panels (C, D) show the particulate sulfur formation rates and the pH for both aerosol types. High measured HMS and nighttime sulfate indicate an aerosol phase that remains externally mixed (e.g., separate sulfate and organic particles) due to the heterogeneous source distribution and short residence time in the PSL. Aerosol self-acidification is prevented by continuous renewal of the aerosol through emission and export from the PSL, and sustains sulfur processing throughout the entire pollution event. Enhanced daytime production can transiently reduce the pH.

We use PACT-1D to assess if known multiphase chemistry in aerosol particles can explain secondary particulate sulfate and HMS observations in the PSL air mass, which is, as shown above, strongly influenced by mixing with background air. The poorly constrained aerosol mixing state, nonideal chemistry in a high-salinity matrix^11,21,33,34^ and uncertain partitioning coefficients and reaction rates at cold temperatures introduce uncertainties in the model that are challenging to quantify. Here, we want to focus on process understanding and the relevance of the interplay between atmospheric mixing and chemical multiphase kinetics, which is often ignored in PSL chemistry studies. Therefore, and in order to avoid further complication, we base our study on a chemical mechanism for dilute solutions and discuss the impact of the respective uncertainties on our results.

Sulfate isotope analysis indicates that H_2_O_2_ is an important sulfate oxidant throughout ALPACA.^22^ However, the HO_2_ levels in the PSL are too low to produce enough H_2_O_2_ in the gas phase (Figure 2B). It has been shown recently that in-particle photolysis of BrC can produce significant amounts of H_2_O_2._ ^14,27^ This was added to our chemical mechanism based on BrC observations and photolysis frequencies measured in the laboratory (see Supporting Information). In addition, we include primary particulate sulfate emissions to match measured primary sulfate levels (see Figure S5).

In our first simulation, aerosol containing primary sulfate and BrC is internally mixed, i.e., the chemical composition of all PSL particles is the same. The resulting daytime secondary sulfate levels agree with the measurements, while nighttime sulfate and HMS are underestimated (Figure 3A,B). In the internally mixed case, the major sulfate formation pathways are the aerosol reactions of S­(IV) (HSO_3_^–^ + SO_3_^2–^) with NO_2_, H_2_O_2_ from in-particle BrC photolysis, and peroxynitric acid (PNA, Figure 3C).

The aerosol pH (Figure 3C) is controlled by the availability of ammonia. When the PSL is present, ammonia emissions accumulate near the surface and buffer the pH of the internally mixed aerosol to values around 5, while during times of larger vertical mixing, and, thus, low ammonia concentrations, the pH drops to values around 0 due to the high amounts of unneutralized primary sulfate. The two pH levels agree well with equilibrium model outputs for the same time period (ref 7 and Figure S11). This indicates the accuracy of the EPA reported ammonia emission rates used in our simulations, which, due to the absence of ammonia measurements during ALPACA, are among the main model uncertainties.

Enhanced sulfate production and the partitioning of nitric acid into the aerosol phase cause a notable transient decrease of the aerosol pH during daytime (Figure 3C). This pH decrease is counteracted by the continuous renewal of the PSL aerosol through removal of acidic particles from the PSL and new particle and ammonia emissions. The renewal process explains the stability of the aerosol pH, which otherwise would rapidly drop, slowing down most secondary sulfur formation pathways significantly.

As argued above, mixing also limits the prevailing pollutant concentrations through their limited residence time in the PSL. For example, at a pH of 5 significant HMS production is commonly expected.^7^ However, the continuous pollutant removal from the PSL prevents HMS from accumulating to the measured levels. The same applies to nighttime secondary sulfate.

While modeling internally mixed aerosol often provides a representative macroscopic view on aerosol chemistry, shallow PSL scenarios with mixing time scales on the order of 1 h and heterogeneous pollutant source distributions are unlikely to host an entirely internally mixed aerosol phase.^10,15^ Aerosols from different sources, e.g., sulfate particles from heating oil combustion and organic PM from biomass burning likely remain, to some extent, in separate particles of an externally mixed aerosol phase.^10,15^ In order to quantify the influence of the aerosol mixing state on the formation of sulfur aerosol in the PSL, we perform a separate model run, in which the model aerosol phase represents only one main component of the externally mixed aerosol. We chose to simulate the chemistry in ALW that is associated with the abundant organic aerosol in the PSL and has not yet mixed with primary sulfate particles. The model implementation is identical to the internally mixed aerosol except that the primary sulfate is not added to the model ALW; i.e., the particles contain the organic material and the secondary inorganic species formed in situ. For simplicity, the amount of ALW is assumed to be the same as in the internally mixed case. This is justified by (a) thermodynamic model estimates of ALW associated with organic aerosol on the order of 30% [ref 7 and Figure S4] and (b) single particle analysis indicating that secondary sulfate during ALPACA is associated with biomass burning organic particles,^15^ causing further ALW uptake. Because the primary sulfate aerosol will still be present in the PSL, we parametrize its effect on the available ammonia, i.e., for each emitted sulfate molecule we remove two ammonia molecules from the emissions that would be consumed by primary sulfate neutralization at the pH of 5. This model run is referred to as “low-sulfate ALW” and represents one end-member of the PSL aerosol mixture we consider particularly relevant for PSL chemistry.

The pH of the low-sulfate ALW is about 0.5–0.8 higher than that of the internally mixed aerosol, resulting in substantially higher particulate sulfur production rates. These lead to a better agreement of measured and modeled nighttime sulfate and explain the presence of HMS in the PSL (Figure 3), pointing toward the significance of the aerosol mixing state for the composition of shallow PSLs.

The remaining small discrepancy between model and measurements of secondary sulfur aerosol are well within measurement and model uncertainties, e.g., due to slight underestimation of ammonia emissions. Furthermore, isotopic composition measurements indicate that H_2_O_2_ dominates sulfate formation during the day,^22^ while our simulations show a larger contribution of oxidation by NO_2_. This could be explained by a slight underestimation of the BrC photooxidant production and unknown rate constants and partitioning coefficients at low temperature and high ionic strength. Recent studies propose strong changes in chemical processing rates in small aerosol particles due to high ionic strength or surface effects. For instance, sulfate production by both NO_2_ and H_2_O_2_ is expected to increase by up to 2–3 orders of magnitude based on laboratory experiments.^20,21^ These rate enhancements will not directly translate into real-world sulfate production rates under shallow PSL conditions, because sulfate production by H_2_O_2_ in arctic winter scenarios will be limited by in-particle photooxidant (H_2_O_2_) production. The enhancement of the rate of sulfate formation via S­(IV) + NO_2_ will rapidly become self-limited because it lowers the aerosol pH (see discussion in Supporting Information and Figure S6). Consequently, proposed rate enhancements due to high ionic strength require reevaluation considering environment specific aspects, such as the availability/accumulation of photoactive PM and the aerosol pH in shallow and cold PSLs, which, as shown above, are controlled by atmospheric mixing.

PSL aerosol chemistry remains challenging to quantify, particularly due to the large uncertainties in physicochemical processes at low temperature and high ionic strength. Nevertheless, our integrated transport and chemistry modeling reveals the crucial importance of the exchange of the PSL with background air for understanding and quantifying PSL chemistry. We showed that particle-type-specific simulations can help to assess the influence of an externally mixed aerosol, which is expected due to short pollutant residence times and spatially heterogeneous sources. An organics derived low-sulfate component of the externally mixed aerosol can explain the presence of high-pH ALW, which allows for the observed fast sulfur processing in the PSL. Moreover, the control of the particle pH through continuous aerosol renewal and the short residence times limiting pollutant concentrations is essential to explain the PSL composition over the course of a multiday pollution event.

### The Response of the PSL Composition to Emission Reductions

Integrated simulations of the mixing of PSL air with ambient air and kinetic multiphase chemistry provide a comprehensive and quantitative explanation for the observed high secondary inorganic pollutant levels in cold and shallow PSLs (Figure 4A). Both pollutant concentrations and their production pathways in the PSL are substantially controlled by atmospheric mixing. On that basis, we will analyze different mitigation measures by reducing specific pollutant emission rates in the model (organic PM, sulfur, and NO_X_) and evaluating the response of average near-surface secondary pollutant concentrations during the cold polluted event studied above. Here we consider emission reductions to fractions of 0.5 and 0.2 of the initial emission rates and their influence on chemical production rate averages between Jan. 30 and Feb. 2 2022 (see Figures S7, S8, S9, for more details).

**Figure 4:**
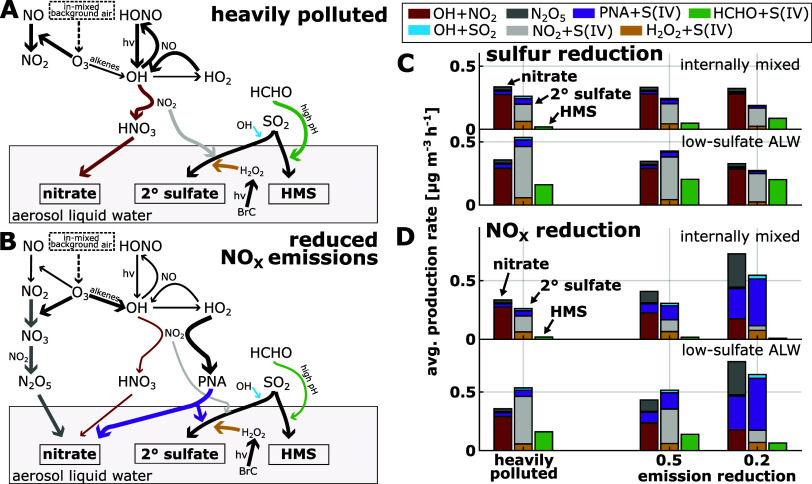
Summary of the major chemical processes (arrow thickness roughly indicates the rate) under heavily polluted conditions (A) and when NO_X_ emissions are reduced (B). Panels (C, D) show simulated average nitrate, secondary sulfate, and HMS formation rates at 3 m above ground between Jan. 30 to Feb. 2, and how they change with reductions of sulfur and NO_X_ emissions in the internally mixed aerosol and the low-sulfate ALW, respectively. Reduction of SO_2_ and primary sulfate emissions (e.g., reduction of sulfur content in heating oil, C) will reduce overall sulfate concentrations in the PSL, because of the large contribution of primary emissions to total particulate sulfur. However, because less NH_3_ is consumed by primary sulfate neutralization, the aerosol pH increases, thus enhancing secondary sulfate and HMS formation, which offsets the effect of SO_2_ concentration reductions. Reduced NO_X_ and HONO emissions (e.g., road traffic, D) during a strong PSL event enhance average secondary nitrate and sulfate levels, because higher HO_2_ levels increase PNA formation and the larger availability of O_3_ allow N_2_O_5_ production (B).

A reduction of organic aerosol emissions, which are, e.g., related to residential wood burning, would also reduce the low-sulfate ALW component of the aerosol and thus slow down sulfate and HMS formation in the aerosol (see Figure S10). A reduction of sulfur emissions, e.g., by reducing the sulfur content of heating oil, will lower the concentrations of primary sulfate aerosol and SO_2_ in the PSL. However, our model results show that, because less ammonia is consumed by primary sulfate neutralization, the aerosol pH increases, which sustains similar particulate sulfur production rates, despite the reduced SO_2_– and thus S­(IV)– concentrations (Figures 4C and S7). Organic PM and sulfur reduction scenarios hardly affect nitrate levels, because nitrate originates from nitric acid, which is formed in the gas phase and entirely partitions into the aerosol.

The response of the PSL composition to reductions of NO_X_ and associated HONO emissions (mostly from road traffic) is much more complex (Figures 4B,D, S8, and S9). We observe two major changes in the chemical system. (1) Reduced NO levels increase the availability of O_3_ entrained from the background. This increases the rate of the reactions of O_3_ with alkenes, forming OH, and O_3_ with NO_2_, which starts producing significant amounts of nitrate radicals and N_2_O_5_. The latter efficiently hydrolyzes to form particulate nitrate. (2) Reduced NO also cause higher levels of HO_2_, which react with NO_2_ to form PNA. PNA then forms substantial nitrate and sulfate in the particle phase. Strikingly, these changes in the oxidation regime caused by reducing NO_X_ can lead to a net increase of average particulate nitrate and sulfate formation in the PSL, despite precursor, i.e., NO_2_, reductions (Figure 4B).

Naturally, these considerations are influenced by the above-discussed uncertainties in particle phase sulfur chemistry. If the pH-independent H_2_O_2_ sulfate formation pathway was dominating sulfate production, sulfur emission reductions would reduce secondary sulfate more effectively. For the NO_X_ reduction scenario sulfate levels would be even higher because H_2_O_2_ oxidation–unlike NO_2_ oxidation–does not depend on the concentration of NO_X_.

In conclusion, we could explain the unique and complex chemistry underlying the pollutant composition of cold and shallow PSLs in Fairbanks. Pollutant concentrations and their chemistry is governed by persistent mixing of the PSL air with the clean background atmosphere on a time scale on the order of 1 h, indicating rapid formation of secondary species. High HONO concentrations cause high levels of HO_X_ in the daytime PSL, driving the formation of nitric acid, which partitions to the aerosol phase and forms nitrate. Continuous mixing with the clean background air mass explains the observed diurnal cycle in nitrate observations and the stability of the aerosol pH required for the observed persistent rapid particulate sulfur formation throughout the multiday pollution event. Furthermore, we show that an externally mixed aerosol in shallow PSLs can contain high-pH particles providing the observed rapid formation of sulfate and HMS. This complex multiphase chemistry-transport system in the PSL reacts in unexpected ways to the reduction of NO_X_ emissions by increasing PSL particulate sulfate and nitrate amounts. Overall, we showed that cold and shallow PSLs are by no means “stagnant” and that a quantitative understanding of PSL air pollution and the development of effective mitigation strategies requires an accurate description of coupled mixing and multiphase chemistry with vertically highly resolved air quality models. Although our case study examined a rather extreme PSL event, the role of atmospheric mixing will be similarly important in many other environments with locally confined pollution.

## Supplementary Material



## Notes

Additional detailed description of key PACT-1D model aspects, gas- and aqueous-phase chemical mechanisms, equilibria and Henry’s law partitioning parameters (Tables S1–S5); comparison of modeled and measured trace gas concentrations, aerosol liquid water content, and primary sulfate (Figures S1–S5); additional sensitivity analyses (Figures S6–S11) (PDF)

## ∇

Institute of Environmental Physics, Heidelberg University, 69120 Heidelberg, Germany
