# Supplementary material for: Atmospheric Mixing Controls Air Pollution Chemistry and Mitigation Efficacy in High-Latitude Cities
Source: Environ Sci Technol. 2026 Jul 23;60(30):21104–12. doi: 10.1021/acs.est.6c00181 (PMC13449895; doi:10.1021/acs.est.6c00181)
Supplement: Supplementary file 1 [file es6c00181_si_001.pdf]

Supporting Information for: Atmospheric mixing controls air pollution chemistry and mitigation efficacy in high-latitude cities

Jonas Kuhn[1,*,a], Laura M. D. Heinlein[2], Meeta Cesler-Maloney[3], Michael O. Sunday[2], Fangzhou Guo[4,5], James H. Flynn[4], Allison Moon[6], Becky Alexander[6], William R. Simpson[3], Cort Anastasio[2], and Jochen Stutz[1]

[1]Department of Atmospheric and Oceanic Sciences, University of California, Los Angeles, CA 90095, USA [2]Department of Land, Air and Water Resources, University of California, Davis, CA 95616, USA [3]Department of Chemistry and Biochemistry, University of Alaska, Fairbanks, AK 99775, USA [4]Department of Earth and Atmospheric Sciences, University of Houston, Houston, TX 77204, USA [5]Aerodyne Research Inc., Billerica, MA 01821, USA [6]Department of Atmospheric Sciences, University of Washington, Seattle, WA 98195, USA

[a]now at: Institute of Environmental Physics, Heidelberg University, 69120 Heidelberg, Germany

[*]Correspondence: jonaskuhn-sci@posteo.de

Contents

S1 Platform for Atmospheric Chemistry and Transport in one Dimension, PACT-1D **S3**

S1.1 Comparing spatially averaged model results with point/line measurements S3

S1.2 The gas-phase chemistry and emissions of NO_X S3

S1.3 Nitrous acid in Fairbanks S3

S1.4 Multi-phase chemistry in PACT-1D S4

S1.4.1 Brown carbon (BrC) photolysis and the formation of aqueous H_2O_2 . . . S4

S1.4.2 The influence of a high ionic strength deliquescent aerosol phase S4

List of Figures

S1 Different boundary conditions (temperature, wind speed, photolysis frequency of HONO), model outputs (SO_2 mixing ratio, see also Fig. S2), and derived quantities (pollutant residence time in the PSL and the ratio of deposition to advective export). PM1 data is from [9] S13

S2 Trace gas measurements at 3m above ground (black dots) at the CTC building in Fairbanks [see 20]. The drawn lines show the simulated concentration in a model run without heterogeneous chemistry (red) and for the organic aerosol implementation (yellow). The model runs hardly differ and represent the measurements well. NO_X, O_3, CO, and SO_2 concentration are not influenced by multiphase processing in the PSL. S14

S3 Trace gas measurements along a 1.2 km long light path 12-17m above ground (black dots) in downtown Fairbanks [see 20, 21]. The drawn lines show the simulated concentration in a model run without heterogeneous chemistry (red) and for the organic aerosol implementation (yellow). The model runs agree well with the measurements and the measured trace gases are hardly influenced by aerosol chemistry. This for instance excludes significant HONO production from the aerosol chemistry considered in this study. S15

S4 Aerosol liquid water content modeled by [3] based on the assumption of an internally mixed aerosol phase in thermodynamic equilibrium (TE), with (drawn) and without organics (drawn) using two different TE models. The liquid water content in our model runs (red line), which is informed by particle number and radius measurements (see also Fig. S1) agrees well and highlights that our assumptions on the aerosol liquid water content generally agree with TE models. Note that deviations are expected due to uncertainties and different assumptions on the aerosol mixing state. S16
S5 Sulfate mass concentration in PACT-1D due to primary sulfate emissions (red line). Measured primary sulfate (black crosses) by [14]. S16
S6 Fig. 3 of the article alongside model results with the aqueous chemical mechanism modified according to recent findings in high ionic strength influences (as indicated in Tab. S5). Increased HMS formation rates in high ionic strength aerosol as found by [27] would cause too high HMS levels (middle panel). The right panel shows a model run with all high ionic strength modifications except that of the HMS formation. S17
S7 Sulfate and HMS formation after the reduction of sulfur (particulate sulfate and SO_2) emissions in internally mixed aerosol (middle) and the low sulfate ALW component (right panel) of an externally mixed aerosol. The reduction of particulate sulfate emissions cause higher levels of ammonia in the gas phase and thus increases the aerosol pH. The corresponding enhanced rates of HMS formation and sulfate oxidation via NO_2 partly compensate the precursor (SO_2) emission reduction. S17
S8 HO_X chemistry and nitrate formation for the model runs with reduced NO_X (and associated HONO) emissions (see Fig. 4 of main article). Nitrate formation during night increases due to N_2O_5 chemistry and PNA formation. Nighttime HO_X is formed predominantly by the reaction of O_3 with alkenes. S18
S9 Rates corresponding to the average values shown in Fig. 4E in the article. In both types of aerosol, the reduction of NO_X leads to enhanced production of sulfate. Aerosol pH gets lower through enhanced production, favoring the PNA sulfate oxidation pathway and reducing HMS formation S18
S10 Effect of the reduction of organic PM emissions (e.g. residential wood burning): The low sulfate, higher pH ALW component of the externally mixed aerosol would be reduced, and thus, in-particle formation, particularly the processes that require high pH, would decrease almost linearly. S19
S11 The high mode of the pH of the internally mixed aerosol derived from thermodynamic equilibrium models during the study period by Campbell et al., 2024 ([3], red crosses) agrees well with our kinetically modeled pH for the internally mixed aerosol case when using the EPA-reported ammonia emissions (solid black line). The dashed and dotted lines illustrate the sensitivity of aerosol pH to ammonia emissions, corresponding to halved and doubled emissions, respectively. The good agreement indicates that the EPA-reported ammonia emission rates are accurate. S19

List of Tables

S1 measured boundary conditions constraining PACT-1D during ALPACA 2022 . . S9
S2 Formalism approximating multi-phase chemical processes in PACT-1D. D: molecular diffusion coefficient, r_{eff}: effective particle radius, α accommodation coefficient, $\bar{v}$: mean molecular velocity, Θ_{aq}: surface to volume ratio of a particle; w_{aq}: aerosol liquid phase content, Γ: ionization rate, high enough to assure ionic equilibrium, H_s^{cc}: unitless Henry's law coefficient, K_{eq}: ionic equilibrium constant S10

S3 Gas–aqueous phase partitioning parameters.
Data according to [17] (Henry's law constants, henrys-law.org) and [23] (accommodation coefficients). Note that we use the effective Henry's law for HCHO, i.e., HCHO = HCHO + $H_2C(OH)_2$ in the aqueous phase. Aqueous phase rate constants of reactions with HCHO (Tab. S5) account for that.
T: temperature in K; T_0 = 298 K, α: accommodation coefficient; $H(T) = H_0\, e^{-\Delta_{sol}H/R\,(T^{-1}-T_0^{-1})}$; $-\Delta_{sol}H$: enthalpy of dissolution. S10
S4 Aqueous phase ionic equilibria.
Data from [23] and references therein, Equilibrium 1 (water) and 6 (ammonia) have been recalculated based on the Active Thermochemical Tables (atct.anl.gov, last access 11/04/2025).
T: temperature in K; T_0 = 298 K; $K(T) = K_0\, e^{-\Delta H/R\,(T^{-1}-T_0^{-1})}$ S11
S5 Aqueous phase chemical mechanism.
T: temperature in K; T_0 = 298K; R = 8.3 $J\,K^{-1}\,mol^{-1}$; References are given only when deviating from [23] S12

S1 Platform for Atmospheric Chemistry and Transport in one Dimension, PACT-1D

S1.1 Comparing spatially averaged model results with point/line measurements

In our model setup we assume horizontal homogeneity of all simulated processes across the entire 4x4 km area of focus in downtown Fairbanks. This is a simplification for the urban environment, which encompasses heterogeneity in the distribution of pollutant sources. The reported gas emission rates used in the model are spatially averaged over the model domain. The measurements, on the other hand, represent the concentration at a single location [CTC site 20] and averaged along a 1.2km-long light path [Long-Path Differential Optical Absorption Spectroscopy, see 20, 21, for details]. Despite the potential uncertainty introduced by the mismatch of spatial scales, the concentrations of the gas species measured by both techniques (NO_2, O_3, SO_2) agree remarkably well, which indicates that - in most of the time during inversion events - emitted pollutants are distributed relatively homogeneously within the surface layer (see Fig. S2 and S3). The benefits from reducing complexity of transport in our model, i.e. higher transparency and analyzability, lower run-times, and ability to account for detailed chemistry, justify the use or a one-dimensional model. Features that would potentially be captured by 3-dimensional chemistry and transport models would not influence the results of our work.

S1.2 The gas-phase chemistry and emissions of NO_X

Figure S2 shows point measurements of trace gas mixing ratios 3 m above the ground at the CTC site, together with the corresponding model results. Reported temporally resolved NO_X emissions were linearly scaled (factor of 2.3 for NO and 3 for NO_2). While [2] found similar discrepancies in total NO_X in their model and used a temperature dependent correction, linear scaling is sufficient for the limited period we look at, where temperature is permanently low. After the adjustment (red/yellow line in Fig. S2) we find excellent agreement with all measured species. By plotting a model run with and without multi-phase chemistry (which almost perfectly overlap), we can rule out any influence of heterogeneous chemistry on the measured gas-phase concentrations within the PSL.

S1.3 Nitrous acid in Fairbanks

Figure S3 shows the measured trace gas mixing ratios between 12 and 17 m above the ground averaged over the light path of the LP-DOAS instrument [see 20, 21, for details]. We assume that

HONO emissions are linked to NO_X emissions via a constant factor of 1.2 %. While this leads to good agreement between modeled HONO and measurements, the factor of 1.2 % is at the high end of the range of reported HONO to NO_X ratios for vehicle emissions [e.g. 10]. The comparison to a model run without heterogeneous chemistry (i.e., with gas-phase chemistry only, red line in Fig. S3), implies that known aerosol chemistry does not impact the HONO concentrations in the polluted boundary layer significantly. Among others, our mechanism includes nitrate photolysis, NO_2 hydrolysis, and sulfur oxidation by NO_2 as possible heterogeneous HONO sources. Also the reported enhanced reaction rates in small high ionic strength aerosol particles, e.g. for NO_2 hydrolysis [7] or sulfur oxidation by NO_2 [12], do not significantly influence the modeled HONO levels. Future studies should also include the influence of heterogeneous snow chemistry as it could influence the surface layer HONO levels by providing a large surface area and additional liquid phase for reactions [11]. Here, we focus on understanding the consequences of the measured high HONO levels during the ALPACA campaign, for which an exact knowledge of the sources of HONO is not required. If in the sensitivity studies HONO was not reduced alongside NO_X emissions, nitrate levels would increase even more (see Fig. 4 in the article).

S1.4 Multi-phase chemistry in PACT-1D

Tab. S2 describes the kinetic representation of gas-particle partitioning in PACT-1D. We use as kinetic implementation of Henry's law with mass transfer limitation according to [18] using a static particle radius of 150 nm [16], accommodation coefficients α [23, and references therein] and Henry's law coefficients reported by [17]. The species relevant for this study are always close to Henry's law equilibrium and, thus, are not mass transfer limited. Ionic equilibria are assumed to establish instantaneously, which is implemented by using a fast forward reaction rate constant. The kinetic aqueous chemical mechanism is shown is Tab. S5.

S1.4.1 Brown carbon (BrC) photolysis and the formation of aqueous H_2O_2

We assume that aerosol liquid water phase contains light absorbing BrC, which partakes in photochemical H_2O_2 formation [22]. We use the spectrally resolved downwelling actinic flux measurements I_λ [photons $cm^{-2}\,s^{-1}$] and the BrC mass absorption cross sections $\sigma_{m,\lambda}$ in [$cm^2\,g^{-1}$] normalized to the dissolved organic carbon amount measured by [8] to calculate the photolysis frequency

$$j_{BrC,abs} = \int_{310nm}^{550nm} \mathrm{d}\lambda \, I_\lambda \, (a+1) \, \sigma_{m,\lambda} \, M_C \, N_A^{-1} \, \kappa$$

with the molar mass of carbon M_C=12.011 g mol^{-1}, the Avogadro constant N_A, the albedo a, and a unitless factor κ = 2.5 accounting for optical confinement inside particles [5, 8]. The photolysis frequency for the photolytic production of H_2O_2 is then given by

$$j_{BrC,H_2O_2} = j_{BrC,abs} \, \phi_{H_2O_2}$$

with the quantum yield ϕ for H_2O_2 production reported by [22].

S1.4.2 The influence of a high ionic strength deliquescent aerosol phase

In our main work, we assume dilute aerosol liquid water, recognizing that high ionic strength can impact aerosol chemistry and gas-exchange. Our choice of using dilute aqueous chemistry avoids the current uncertainties in quantifying ionic strength effects and allows us to build our analysis on known chemistry. For instance, recent literature finds opposing effects of ionic strength on HMS formation [26, 27], proposing either an order of magnitude decrease or several orders of magnitude increase of the formation rate. Our results can serve as a base for resolving such issues, which is however beyond the scope of this work.

A rudimentary sensitivity study helps identifying central problems and enables a first assessment of the relevance of high ionic strength effects: Recent literature highlights substantially different (generally faster) chemistry in high ionic strength deliquescent aerosol when compared to the well constrained chemistry in the larger dilute cloud particles. During the polluted ALPACA period studied here, the ionic strength of a PSL particle based on the measured particulate sulfate, nitrate, HMS, and ammonium would peak at around 20M, which is common for urban haze scenarios. When assuming externally mixed ALW without primary sulfate the ionic strength is about 5.5M. Several reactions in our dilute aqueous chemical mechanism have been found to proceed substantially faster in high ionic strength deliquescent aerosol, including NO_2+NO_2 [7], HSO_3^-+O_3 [25], HSO_3^-+H_2O_2 [13], SO_3^{2-}+NO_2 [12], and HMS formation [27]. Most of these studies focus on a single chemical processing pathway and quantify the overall effect on an effective chemical process, including partitioning between gas and aerosol phase, ionic dissociation, and aqueous phase chemical reaction. All of the individual steps might, however, be affected differently by high ionic strength. In order to assess the effect on our model results, we assigned the proposed enhancement in chemical processing to the kinetic aqueous rate constant of the individual processes. We assume a higher ionic strength (ca. 20M) in our aerosol, in order to assess an upper limit of the influence of the reported high ionic strength reactions. The modifications are listed in Tab. S5. Fig. S6 shows the results for two model runs; (1) all high ionic strength modifications (2) all high ionic strength modifications except the modification for HMS.

If we use the reported increased HMS production rate constant of [27], our simulations strongly overestimate HMS levels, demanding further, refined laboratory studies particularly focusing on solubility and reaction rate constants at low temperatures.

When ignoring the HMS modification, the high ionic strength model run produces reasonable - though somewhat higher than measured - secondary sulfate amounts in the PSL. We see no effect of the modified O_3 reaction, as O_3 concentrations are still too low. The production of sulfate via H_2O_2 remains unchanged by the orders of magnitude increase in the rate constant, because the process is limited by the availability of H_2O_2, formed by BrC photolysis. The enhanced sulfate production from SO_3^{2-}+NO_2 accounts for most of the secondary sulfate enhancement. However, larger sulfate production rates lower the pH. As a result, despite an increase of the reaction rate constant by several orders of magnitude, sulfate levels only change by a factor of ca. 2-3. The replacement of low pH aerosol with fresh, higher pH particles, as explained in the article, controls secondary aerosol production in the PSL.

Looking at these individual processes helps understanding and partially explains differences between simulations and observations. However, a comprehensive study of the influence of high ionic strength would need to include all chemical processes, e.g. PNA formation, for which we could not find literature data, and to assign high ionic strength effects to the individual steps of partitioning, ionization, and reaction. By providing a kinetic treatment of multi-phase chemistry, PACT-1D can help inform future integrated laboratory studies.

References

[1] Scott D. Boyce and Michael R. Hoffmann. Kinetics and mechanism of the formation of hydroxymethanesulfonic acid at low pH. *The Journal of Physical Chemistry*, 88(20):4740–4746, September 1984. ISSN 0022-3654, 1541-5740. doi: 10.1021/j150664a059.

[2] Natalie Brett, Steve R. Arnold, Kathy S. Law, Jean-Christophe Raut, Tatsuo Onishi, Brice Barret, Elsa Dieudonné, Meeta Cesler-Maloney, William Simpson, Slimane Bekki, Joel Savarino, Sarah Albertin, Robert Gilliam, Kathleen Fahey, George Pouliot, Deanna Huff, and Barbara D'Anna. Estimating Power Plant Contributions to Surface Pollution in a Wintertime Arctic Environment. *ACS ES&T Air*, 2(5):943–956, May 2025. ISSN 2837-1402, 2837-1402. doi: 10.1021/acsestair.5c00030.

[3] James R. Campbell, Michael Battaglia Jr., Kayane K. Dingilian, Meeta Cesler-Maloney, William R. Simpson, Ellis S. Robinson, Peter F. DeCarlo, Brice Temime-Roussel, Barbara D'Anna, Andrew L. Holen, Judy Wu, Kerri A. Pratt, Jack E. Dibb, Athanasios Nenes, Rodney J. Weber, and Jingqiu Mao. Enhanced aqueous formation and neutralization of fine atmospheric particles driven by extreme cold. *Science Advances*, 10(36):eado4373, September 2024. ISSN 2375-2548. doi: 10.1126/sciadv.ado4373.

[4] Carol L. Clifton, Nisan. Altstein, and Robert E. Huie. Rate constant for the reaction of nitrogen dioxide with sulfur(IV) over the pH range 5.3-13. *Environmental Science & Technology*, 22(5):586–589, May 1988. ISSN 0013-936X, 1520-5851. doi: 10.1021/es00170a018.

[5] Pablo Corral Arroyo, Grégory David, Peter A. Alpert, Evelyne A. Parmentier, Markus Ammann, and Ruth Signorell. Amplification of light within aerosol particles accelerates in-particle photochemistry. *Science*, 376(6590):293–296, April 2022. ISSN 0036-8075, 1095-9203. doi: 10.1126/science.abm7915.

[6] Vinícius Wilian D. Cruzeiro, Mirza Galib, David T. Limmer, and Andreas W. Götz. Uptake of N2O5 by aqueous aerosol unveiled using chemically accurate many-body potentials. *Nature Communications*, 13(1):1266, March 2022. ISSN 2041-1723. doi: 10.1038/s41467-022-28697-8.

[7] Masao Gen, Haotian Zheng, Yele Sun, Wanyun Xu, Nan Ma, Hang Su, Yafang Cheng, Shuxiao Wang, Jia Xing, Shuping Zhang, Likun Xue, Chaoyang Xue, Yujing Mu, Xiao Tian, Atsushi Matsuki, and Shaojie Song. Rapid hydrolysis of NO_2 at High Ionic Strengths of Deliquesced Aerosol Particles. *Environmental Science & Technology*, 58(18):7904–7915, May 2024. ISSN 0013-936X, 1520-5851. doi: 10.1021/acs.est.3c08810.

[8] Laura M. D. Heinlein, Junwei He, Michael Oluwatoyin Sunday, Fangzhou Guo, James Campbell, Allison Moon, Sukriti Kapur, Ting Fang, Kasey Edwards, Meeta Cesler-Maloney, Alyssa J. Burns, Jack Dibb, William Simpson, Manabu Shiraiwa, Becky Alexander, Jingqiu Mao, James H. Flynn Iii, Jochen Stutz, and Cort Anastasio. Surprisingly robust photochemistry in subarctic particles during winter: Evidence from photooxidants. *Atmospheric Chemistry and Physics*, 25(16):9561–9581, August 2025. ISSN 1680-7324. doi: 10.5194/acp-25-9561-2025.

[9] Amna Ijaz, Brice Temime-Roussel, Benjamin Chazeau, Sarah Albertin, Stephen R. Arnold, Brice Barret, Slimane Bekki, Natalie Brett, Meeta Cesler-Maloney, Elsa Dieudonne, Kayane K. Dingilian, Javier G. Fochesatto, Jingqiu Mao, Allison Moon, Joel Savarino, William Simpson, Rodney J. Weber, Kathy S. Law, and Barbara D'Anna. Complementary aerosol mass spectrometry elucidates sources of wintertime submicron particle pollution in Fairbanks, Alaska, during ALPACA 2022. *Atmospheric Chemistry and Physics*, 25(19): 11789–11811, October 2025. ISSN 1680-7324. doi: 10.5194/acp-25-11789-2025.

[10] Louisa J. Kramer, Leigh R. Crilley, Thomas J. Adams, Stephen M. Ball, Francis D. Pope, and William J. Bloss. Nitrous acid (HONO) emissions under real-world driving conditions from vehicles in a UK road tunnel. *Atmospheric Chemistry and Physics*, 20(9):5231–5248, May 2020. ISSN 1680-7324. doi: 10.5194/acp-20-5231-2020.

[11] Jonas Kuhn, Jochen Stutz, Thorsten Bartels-Rausch, Jennie L. Thomas, Meeta Cesler-Maloney, William R. Simpson, Jack E. Dibb, Laura M. D. Heinlein, and Cort Anastasio. The interplay between snow and polluted air masses in cold urban environments. *Faraday Discussions*, 258:502–520, 2025. ISSN 1359-6640, 1364-5498. doi: 10.1039/D4FD00176A.

[12] Tengyu Liu and Jonathan P. D. Abbatt. Oxidation of sulfur dioxide by nitrogen dioxide accelerated at the interface of deliquesced aerosol particles. *Nature Chemistry*, 13(12): 1173–1177, December 2021. ISSN 1755-4330, 1755-4349. doi: 10.1038/s41557-021-00777-0.

[13] Tengyu Liu, Simon L. Clegg, and Jonathan P. D. Abbatt. Fast oxidation of sulfur dioxide by hydrogen peroxide in deliquesced aerosol particles. *Proceedings of the National Academy of Sciences*, 117(3):1354–1359, January 2020. ISSN 0027-8424, 1091-6490. doi: 10.1073/pnas.1916401117.

[14] Allison Moon, Ursula Jongebloed, Kayane K. Dingilian, Andrew J. Schauer, Yuk-Chun Chan, Meeta Cesler-Maloney, William R. Simpson, Rodney J. Weber, Ling Tsiang, Fouad Yazbeck, Shuting Zhai, Alanna Wedum, Alexander J. Turner, Sarah Albertin, Slimane Bekki, Joël Savarino, Konstantin Gribanov, Kerri A. Pratt, Emily J. Costa, Cort Anastasio, Michael O. Sunday, Laura M. D. Heinlein, Jingqiu Mao, and Becky Alexander. Primary Sulfate Is the Dominant Source of Particulate Sulfate during Winter in Fairbanks, Alaska. *ACS ES&T Air*, page acsestair.3c00023, November 2023. ISSN 2837-1402, 2837-1402. doi: 10.1021/acsestair.3c00023.

[15] Jong Yoon Park and Yin Nan Lee. Solubility and decomposition kinetics of nitrous acid in aqueous solution. *The Journal of Physical Chemistry*, 92(22):6294–6302, November 1988. ISSN 0022-3654, 1541-5740. doi: 10.1021/j100333a025.

[16] Ellis S. Robinson, Meeta Cesler-Maloney, Xinxiu Tan, Jingqiu Mao, William Simpson, and Peter F. DeCarlo. Wintertime spatial patterns of particulate matter in Fairbanks, AK during ALPACA 2022. *Environmental Science: Atmospheres*, 3(3):568–580, 2023. ISSN 2634-3606. doi: 10.1039/D2EA00140C.

[17] Rolf Sander. Compilation of Henry's law constants (version 5.0.0) for water as solvent. *Atmospheric Chemistry and Physics*, 23(19):10901–12440, October 2023. ISSN 1680-7324. doi: 10.5194/acp-23-10901-2023.

[18] Stephen E. Schwartz. Mass-Transport Considerations Pertinent to Aqueous Phase Reactions of Gases in Liquid-Water Clouds. In Wolfgang Jaeschke, editor, *Chemistry of Multiphase Atmospheric Systems*, pages 415–471. Springer Berlin Heidelberg, Berlin, Heidelberg, 1986. ISBN 978-3-642-70629-5 978-3-642-70627-1. doi: 10.1007/978-3-642-70627-1_16.

[19] William Simpson, Meeta Cesler-Maloney, and Ryan Hoskins-Chaddon. Gas and meteorological measurements at the CTC site and Birch Hill in Fairbanks, Alaska, during the ALPACA-2022 field study, 2023.

[20] William R. Simpson, Jingqiu Mao, Gilberto J. Fochesatto, Kathy S. Law, Peter F. DeCarlo, Julia Schmale, Kerri A. Pratt, Steve R. Arnold, Jochen Stutz, Jack E. Dibb, Jessie M. Creamean, Rodney J. Weber, Brent J. Williams, Becky Alexander, Lu Hu, Robert J. Yokelson, Manabu Shiraiwa, Stefano Decesari, Cort Anastasio, Barbara D'Anna, Robert C. Gilliam, Athanasios Nenes, Jason M. St. Clair, Barbara Trost, James H. Flynn, Joel Savarino, Laura D. Conner, Nathan Kettle, Krista M. Heeringa, Sarah Albertin, Andrea Baccarini, Brice Barret, Michael A. Battaglia, Slimane Bekki, T.J. Brado, Natalie Brett, David Brus, James R. Campbell, Meeta Cesler-Maloney, Sol Cooperdock, Karolina Cysneiros de Carvalho, Hervé Delbarre, Paul J. DeMott, Conor J.S. Dennehy, Elsa Dieudonné, Kayane K. Dingilian, Antonio Donateo, Konstantinos M. Doulgeris, Kasey C. Edwards, Kathleen Fahey, Ting Fang, Fangzhou Guo, Laura M. D. Heinlein, Andrew L. Holen, Deanna Huff, Amna Ijaz, Sarah Johnson, Sukriti Kapur, Damien T. Ketcherside, Ezra Levin, Emily Lill, Allison R. Moon, Tatsuo Onishi, Gianluca Pappaccogli, Russell Perkins, Roman Pohorsky, Jean-Christophe Raut, Francois Ravetta, Tjarda Roberts, Ellis S. Robinson, Federico Scoto, Vanessa Selimovic, Michael O. Sunday, Brice Temime-Roussel, Xinxiu Tian, Judy Wu, and Yuhan Yang. Overview of the Alaskan Layered Pollution and Chemical Analysis (ALPACA) field experiment. *Environmental Science & Technology Air*, 1(3): 200–222, 2024. doi: 10.1021/acsestair.3c00076.

[21] Jochen Stutz, Jonas Kuhn, Sol Cooperdock, Sarah Johnson, Fangzhou Guo, James H. Flynn, Meeta Cesler-Maloney, and William R. Simpson. Vertical distribution of pollution trapped by wintertime surface inversions in Fairbanks, Alaska, during the ALPACA experiment. *[preprint]*, March 2026. doi: 10.5194/egusphere-2026-816.

[22] Michael Oluwatoyin Sunday, Laura Marie Dahler Heinlein, Junwei He, Allison Moon, Sukriti Kapur, Ting Fang, Kasey C. Edwards, Fangzhou Guo, Jack Dibb, James H. Flynn Iii, Becky Alexander, Manabu Shiraiwa, and Cort Anastasio. Hydrogen peroxide photoformation in particulate matter and its contribution to S(IV) oxidation during winter in Fairbanks, Alaska. *Atmospheric Chemistry and Physics*, 25(10):5087–5100, May 2025. ISSN 1680-7324. doi: 10.5194/acp-25-5087-2025.

[23] R. Von Glasow and P. J. Crutzen. Model study of multiphase DMS oxidation with a focus on halogens. *Atmospheric Chemistry and Physics*, 4(3):589–608, April 2004. ISSN 1680-7324. doi: 10.5194/acp-4-589-2004.

[24] Peter Warneck. The relative importance of various pathways for the oxidation of sulfur dioxide and nitrogen dioxide in sunlit continental fair weather clouds. *Physical Chemistry Chemical Physics*, 1(24):5471–5483, 1999. ISSN 14639076, 14639084. doi: 10.1039/a906558j.

[25] Chen Yu, Tengyu Liu, Dafeng Ge, Wei Nie, Xuguang Chi, and Aijun Ding. Ionic Strength Enhances the Multiphase Oxidation Rate of Sulfur Dioxide by Ozone in Aqueous Aerosols: Implications for Sulfate Production in the Marine Atmosphere. *Environmental Science & Technology*, 57(16):6609–6615, April 2023. ISSN 0013-936X, 1520-5851. doi: 10.1021/acs.est.3c00212.

[26] Chen Yu, Tengyu Liu, Xuguang Chi, Xin Huang, and Aijun Ding. Ionic Strength Inhibits the Multiphase Reaction Rate between Dissolved SO_2 and HCHO in Aqueous Aerosols: Implications for Sources of Atmospheric Hydroxymethanesulfonate. *Environmental Science & Technology*, 59(30):15900–15906, August 2025. ISSN 0013-936X, 1520-5851. doi: 10.1021/acs.est.5c06148.

[27] Hailiang Zhang, Yongfu Xu, and Long Jia. Hydroxymethanesulfonate formation as a significant pathway of transformation of SO2. *Atmospheric Environment*, 294:119474, February 2023. ISSN 13522310. doi: 10.1016/j.atmosenv.2022.119474.

Table S1: measured boundary conditions constraining PACT-1D during ALPACA 2022

**boundary condition**	**unit**	**Comment**
temperature	K	WRF simulation assimilated to measurements [19, 20]
pressure	$J\,m^{-3}$	WRF simulation assimilated to measurements [19, 20]
vertical diffusivity K_z	$m^2\,s^{-1}$	[21]
aerosol number density	cm^{-3}	[3, 9]
aerosol radius	nm	[16]
trace gas emission rates	$cm^{-3}\,s^{-1}$	inventory provided by USEPA
photolysis frequencies	s^{-1}	[20]
horizontal wind speed	$m\,s^{-1}$	WRF assimilated to measurements [19, 20]
background trace gas conc.	cm^{-3}	static (35 ppb O3, 0.3 ppb SO_2, 250 ppb CO, 0.03 ppb NO,NO_2)

Table S2: Formalism approximating multi-phase chemical processes in PACT-1D.

D: molecular diffusion coefficient, r_{eff}: effective particle radius, α accommodation coefficient, $\bar{v}$: mean molecular velocity, Θ_{aq}: surface to volume ratio of a particle; w_{aq}: aerosol liquid phase content, Γ: ionization rate, high enough to assure ionic equilibrium, H_s^{cc}: unitless Henry's law coefficient, K_{eq}: ionic equilibrium constant

	**uptake to liquid phase**	**aqueous ionic dissociation equilibria**	**aqueous reactions**
schematic exemplary reaction			
	$X \rightleftharpoons X_{aq}$	$X_{aq} \rightleftharpoons Y_{aq}^- + H_{aq}^+$	$X_{aq} + Y_{aq}^- \rightarrow Z_{aq}^- + A_{aq}$
forward rate constant κ including mass transfer limitations to the particle surface κ_{MT} per aqueous volume			
	$\left(\left(\frac{3\,D}{r_{eff}^2}\right)^{-1} + \left(\frac{\bar{v}\,\alpha}{4}\Theta_{aq}\right)^{-1}\right)^{-1}$	-	-
forward rate constant $k_{\rightarrow}$ per atmospheric volume in s^{-1} (1st order), cm^3_{atm} $molec^{-1}s^{-1}$ (2nd order)			
order	1	1	1,2
	$\kappa\, w_{aq}$	Γ	$k_{\rightarrow}^{aq}\, w_{aq}^{1-n}$ see Tab. S5
equilibrium expression used to calculate kinetic backward rate constant $k_{\leftarrow}$			
	$\frac{[X_{aq}]}{[X]} = H_s^{cc}\, w_{aq} = \frac{k_{\rightarrow}}{k_{\leftarrow}}$	$K_{eq}\, w_{aq} = \frac{[Y_{aq}^-][H_{aq}^+]}{[X_{aq}]} = \frac{k_{\rightarrow}}{k_{\leftarrow}}$	-

Table S3: Gas–aqueous phase partitioning parameters.

Data according to [17] (Henry's law constants, henrys-law.org) and [23] (accommodation coefficients). Note that we use the effective Henry's law for HCHO, i.e., HCHO = HCHO + $H_2C(OH)_2$ in the aqueous phase. Aqueous phase rate constants of reactions with HCHO (Tab. S5) account for that.

T: temperature in K; T_0 = 298 K, α: accommodation coefficient; $H(T) = H_0\, e^{-\Delta_{sol}H/R\,(T^{-1}-T_0^{-1})}$; $-\Delta_{sol}H$: enthalpy of dissolution.

#	**species (gas ⇄ aqueous)**	$\boldsymbol{\alpha}$	$\boldsymbol{H_0}$ [mol m^{-3} Pa^{-1}]	$\boldsymbol{-\Delta_{sol}H/R}$ [K]
1	$NO_{2(g)} \rightleftarrows NO_2$	0.0015	1.2×10^{-4}	2400
2	$HONO_{(g)} \rightleftarrows HONO$	0.04	4.8×10^{-1}	4800
3	$HNO_{3(g)} \rightleftarrows HNO_3$	0.5	2.1×10^{3}	8700
4	$HNO_{4(g)} \rightleftarrows HNO_4$	0.1	3.9×10^{-1}	8400
5	$NO_{3(g)} \rightleftarrows NO_3$	0.04	3.8×10^{-4}	0
6	$NH_{3(g)} \rightleftarrows NH_3$	0.06	5.9×10^{-1}	4200
7	$SO_{2(g)} \rightleftarrows SO_2$	0.11	1.3×10^{-2}	2900
8	$O_{2(g)} \rightleftarrows O_2$	0.01	1.2×10^{-5}	1700
9	$O_{3(g)} \rightleftarrows O_3$	0.002	1.0×10^{-4}	2800
10	$HO_{(g)} \rightleftarrows HO$	0.01	3.8×10^{-1}	0
11	$HO_{2(g)} \rightleftarrows HO_2$	0.2	6.8	0
12	$H_2O_{2(g)} \rightleftarrows H_2O_2$	0.077	9.1×10^{2}	6600
13	$CO_{2(g)} \rightleftarrows CO_2$	0.01	3.3×10^{-4}	2400
14	$HCHO_{(g)} \rightleftarrows HCHO$	0.04	3.2×10^{1}	7100
15	$N_2O_{5(g)} \rightleftarrows N_2O_5$	0.93	3.0×10^{-2}	4000
16	$H_2SO_{4(g)} \rightleftarrows H_2SO_4$	0.65	1.0×10^{6}	0

Table S4: Aqueous phase ionic equilibria.

Data from [23] and references therein, Equilibrium 1 (water) and 6 (ammonia) have been recalculated based on the Active Thermochemical Tables (atct.anl.gov, last access 11/04/2025).

T: temperature in K; T_0 = 298 K; $K(T) = K_0\, e^{-\Delta H/R\,(T^{-1}-T_0^{-1})}$

#	equilibrium reaction	K_0	$-\Delta H/R$ [K]
1	$H_2O \rightleftharpoons OH^- + H^+$	1.8×10^{-16}	−6716
2	$CO_2 \rightleftharpoons HCO_3^- + H^+$	4.3×10^{-7}	−913
3	$HCO_3^- \rightleftharpoons CO_3^{2-} + H^+$	4.7×10^{-11}	−1775
4	$SO_2 \rightleftharpoons HSO_3^- + H^+$	1.7×10^{-2}	2090
5	$HSO_3^- \rightleftharpoons SO_3^{2-} + H^+$	6.0×10^{-8}	1120
6	$NH_3 \rightleftharpoons NH_4^+ + OH^-$	1.7×10^{-5}	−463
7	$HNO_3 \rightleftharpoons NO_3^- + H^+$	1.5×10^{1}	0
8	$HONO \rightleftharpoons NO_2^- + H^+$	5.1×10^{-4}	−1260
9	$HNO_4 \rightleftharpoons NO_4^- + H^+$	1.0×10^{-5}	8700
10	$H_2SO_4 \rightleftharpoons HSO_4^- + H^+$	1.0×10^{3}	0
11	$HSO_4^- \rightleftharpoons SO_4^{2-} + H^+$	1.2×10^{-2}	1120
12	$HO_2 \rightleftharpoons O_2^- + H^+$	1.6×10^{-5}	0
13	$HCOOH \rightleftharpoons HCOO^- + H^+$	1.8×10^{-4}	0

Table S5: Aqueous phase chemical mechanism. T: temperature in K; T_0 = 298K; R = 8.3 $J\,K^{-1}\,mol^{-1}$; References are given only when deviating from [23]

#	reaction	rate expression $[\mathrm{M\,s^{-1}}]$ or $[\mathrm{s^{-1}}]$	high ionic strength	comment/ref
reactive oxygen				
O1	$O_3 + OH \rightarrow HO_2$	$1.1 \cdot 10^{8}$		
O2	$O_3 + O_2^- \rightarrow OH + OH^-$	$1.5 \cdot 10^{9}$		
O3	$OH + H_2O_2 \rightarrow HO_2$	$2.7 \cdot 10^{7}\, e^{-1684\,(T^{-1}-T_0^{-1})}$		
O4	$HO_2 + HO_2 \rightarrow H_2O_2$	$9.7 \cdot 10^{5}\, e^{-2500\,(T^{-1}-T_0^{-1})}$		
nitrogen				
N1	$HONO + OH \rightarrow NO_2$	$1.0 \cdot 10^{10}$		
N2	$HONO + H_2O_2 \rightarrow HNO_3$	$4.6 \cdot 10^{3}\, e^{-6800\,(T^{-1}-T_0^{-1})}$		
N3	$NO_3 + OH^- \rightarrow NO_3^- + OH$	$8.2 \cdot 10^{7}\, e^{-2700\,(T^{-1}-T_0^{-1})}$		
N4	$NO_2 + NO_2 \rightarrow HNO_3 + HONO$	$1.0 \cdot 10^{8}\, e^{2900\,(T^{-1}-T_0^{-1})}$		[15]
N5	$NO_2 + HO_2 \rightarrow HNO_4$	$1.8 \cdot 10^{9}$		[24]
N6	$NO_2^- + O_3 \rightarrow NO_3^- + O_2$	$5.0 \cdot 10^{5}\, e^{-6950\,(T^{-1}-T_0^{-1})}$		
N7	$NO_2^- + OH \rightarrow NO_2 + OH^-$	$1.0 \cdot 10^{10}$		
N8	$NO_4^- \rightarrow NO_2^- + O_2$	$8.0 \cdot 10^{-1}$		1st order [24]
N9	$N_2O_5 \xrightarrow{H_2O} 2\,HNO_3$	$4.0 \cdot 10^{7}$		1st order [6]
sulfur				
S1	$HSO_3^- + O_3 \rightarrow SO_4^{2-} + H^+ + O_2$	$3.7 \cdot 10^{5}\, e^{-5500\,(T^{-1}-T_0^{-1})}$	×100	[25]
S2	$SO_3^{2-} + O_3 \rightarrow SO_4^{2-} + O_2$	$1.5 \cdot 10^{9}\, e^{-5300\,(T^{-1}-T_0^{-1})}$	×100	[25]
S3	$SO_3^{2-} + OH \rightarrow SO_3^- + OH^-$	$5.5 \cdot 10^{9}$		
S4	$HSO_3^- + HO_2 \rightarrow SO_4^{2-} + OH + H^+$	$3.0 \cdot 10^{3}$		
S5	$HSO_3^- + O_2^- \rightarrow SO_4^{2-} + OH$	$3.0 \cdot 10^{3}$		
S6	$HSO_3^- + H_2O_2 \rightarrow SO_4^{2-} + H^+$	$5.2 \cdot 10^{6}\, \frac{[H^+]}{[H^+]+0.1\mathrm{M}}\, e^{-3650\,(T^{-1}-T_0^{-1})}$	×100	[13]
S7	$HSO_3^- + NO_2 \rightarrow HSO_4^- + 2\,HONO - NO_2$	$2.0 \cdot 10^{7}$		[4]
S8	$SO_3^{2-} + NO_2 \rightarrow SO_4^{2-} + 2\,HONO - NO_2$	$2.0 \cdot 10^{7}$	×700	[4, 12]
S9	$HSO_3^- + NO_3 \rightarrow SO_3^- + NO_3^- + H^+$	$1.4 \cdot 10^{9}\, e^{-2000\,(T^{-1}-T_0^{-1})}$		
S10	$HSO_3^- + HNO_4 \rightarrow HSO_4^- + NO_3^- + H^+$	$3.1 \cdot 10^{5}$		[24]
S11	$HSO_3^- + HCHO \rightarrow HMS^-$	$2.1 \cdot 10^{10} \cdot T\, e^{\frac{-58.4}{R}}\, e^{\frac{-58.3\cdot 10^3}{RT}}$	$\times 10^4$	Eyring expr., [1, 27]
S12	$SO_3^{2-} + HCHO \rightarrow HMS^- + OH^-$	$2.1 \cdot 10^{10} \cdot T\, e^{\frac{16.7}{R}}\, e^{\frac{-53.4\cdot 10^3}{RT}}$	$k_{S12} \times 10^4$	Eyring expr., [1, 27]
S13	$HMS^- + OH^- \rightarrow SO_3^{2-} + HCHO$	$3.6 \cdot 10^{3}$		

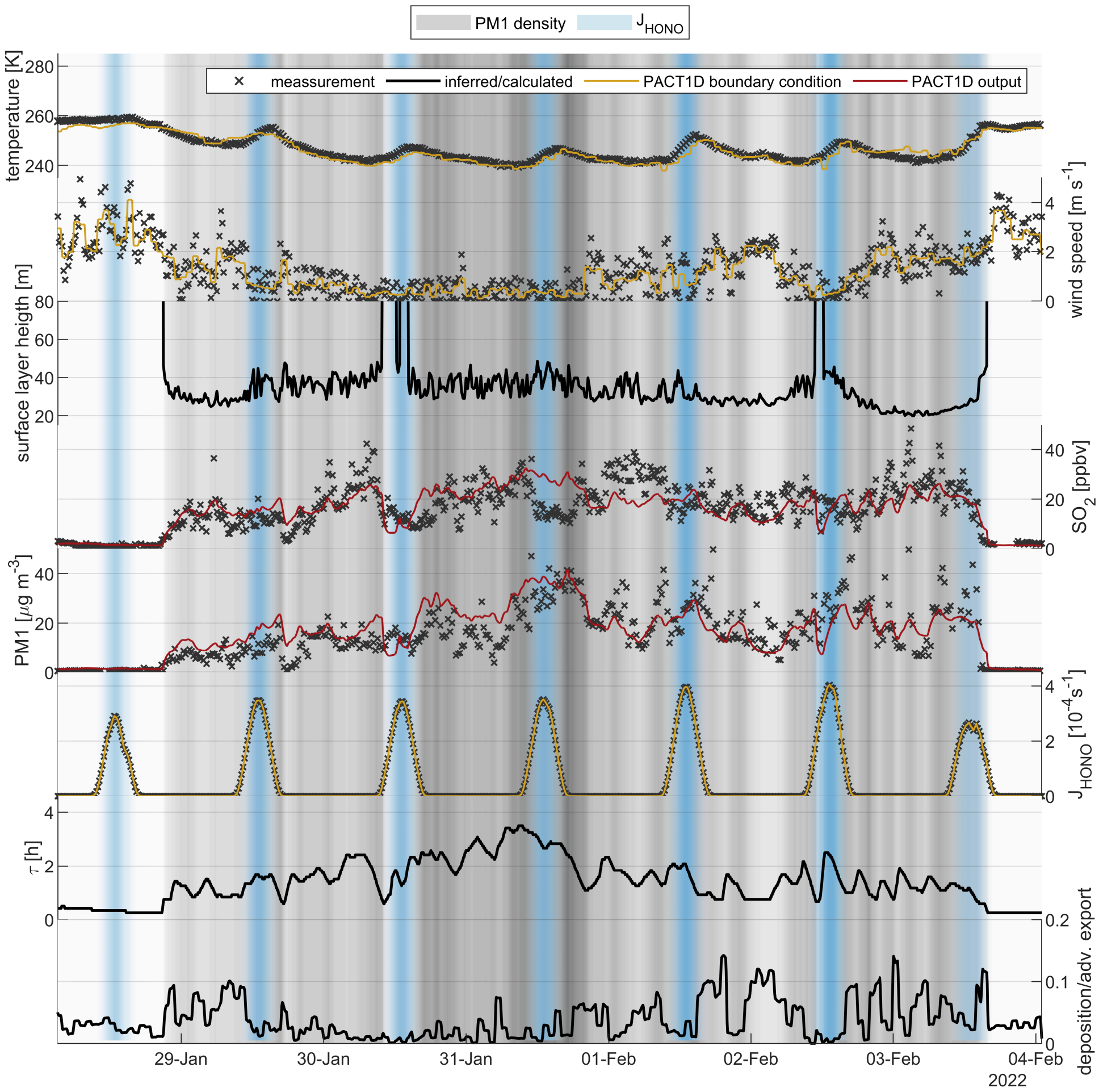

Figure S1: Different boundary conditions (temperature, wind speed, photolysis frequency of HONO), model outputs (SO_2 mixing ratio, see also Fig. S2), and derived quantities (pollutant residence time in the PSL and the ratio of deposition to advective export). PM1 data is from [9]

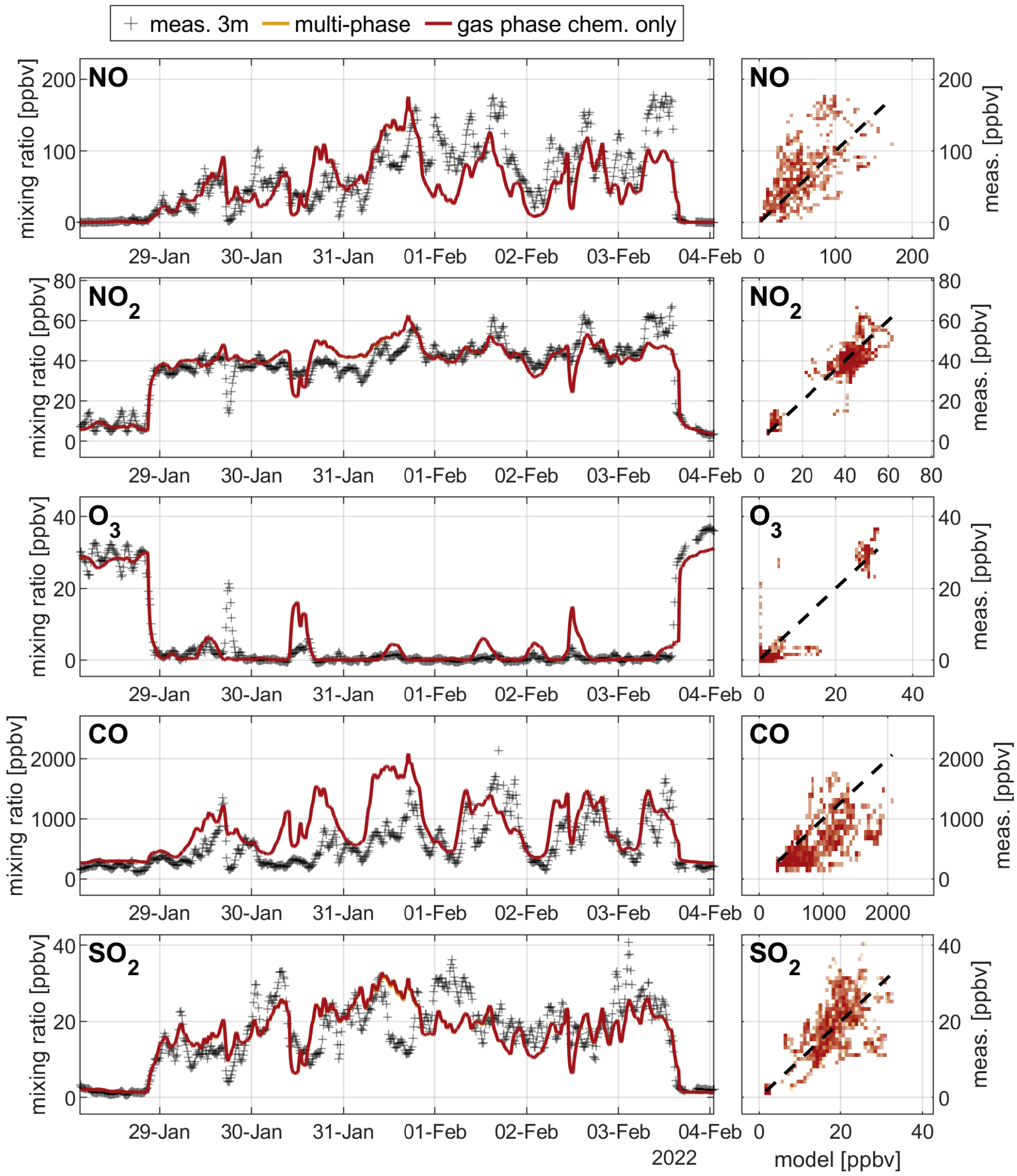

Figure S2: Trace gas measurements at 3m above ground (black dots) at the CTC building in Fairbanks [see 20]. The drawn lines show the simulated concentration in a model run without heterogeneous chemistry (red) and for the organic aerosol implementation (yellow). The model runs hardly differ and represent the measurements well. NO_X, O_3, CO, and SO_2 concentration are not influenced by multiphase processing in the PSL.

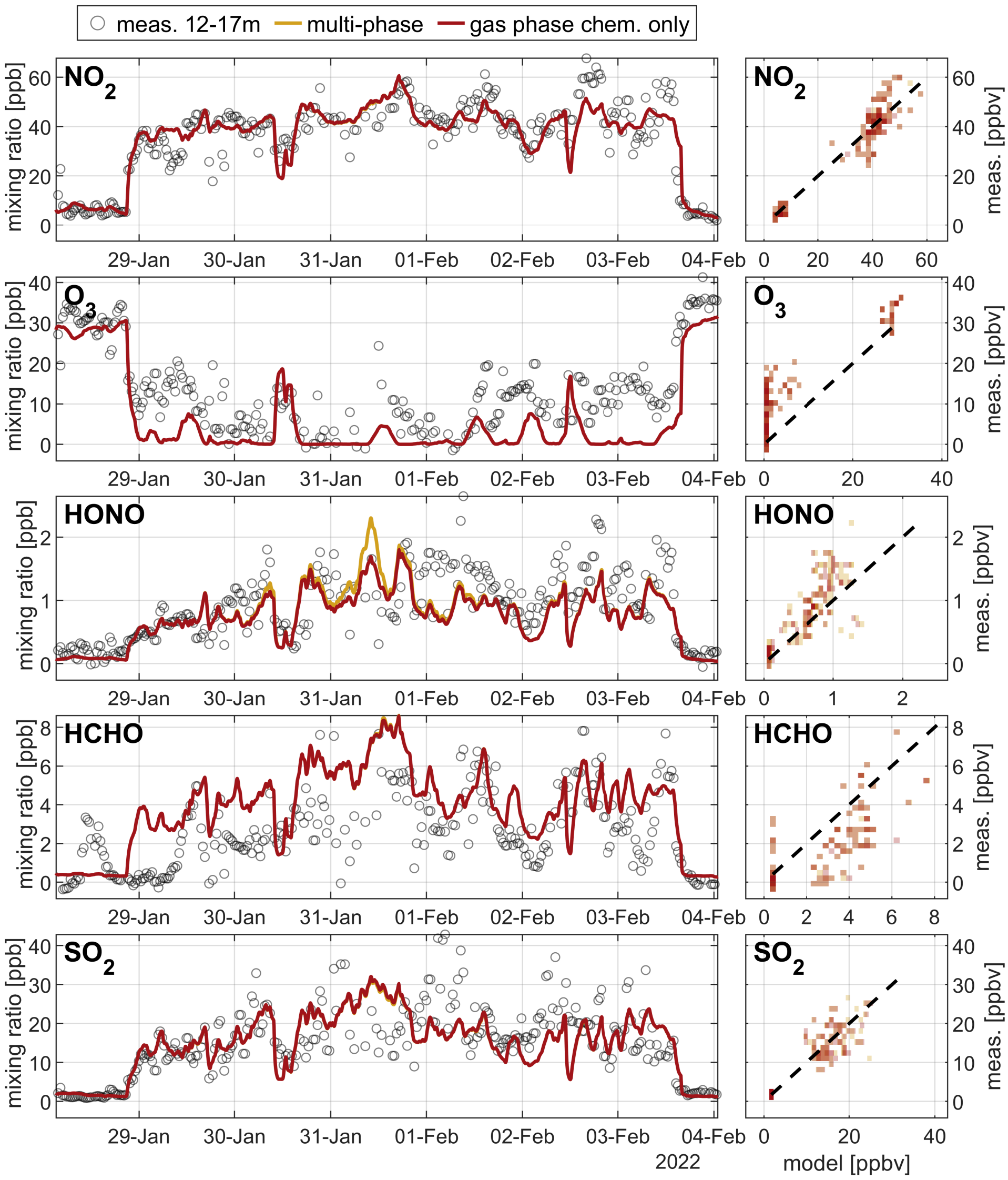

Figure S3: Trace gas measurements along a 1.2 km long light path 12-17m above ground (black dots) in downtown Fairbanks [see 20, 21]. The drawn lines show the simulated concentration in a model run without heterogeneous chemistry (red) and for the organic aerosol implementation (yellow). The model runs agree well with the measurements and the measured trace gases are hardly influenced by aerosol chemistry. This for instance excludes significant HONO production from the aerosol chemistry considered in this study.

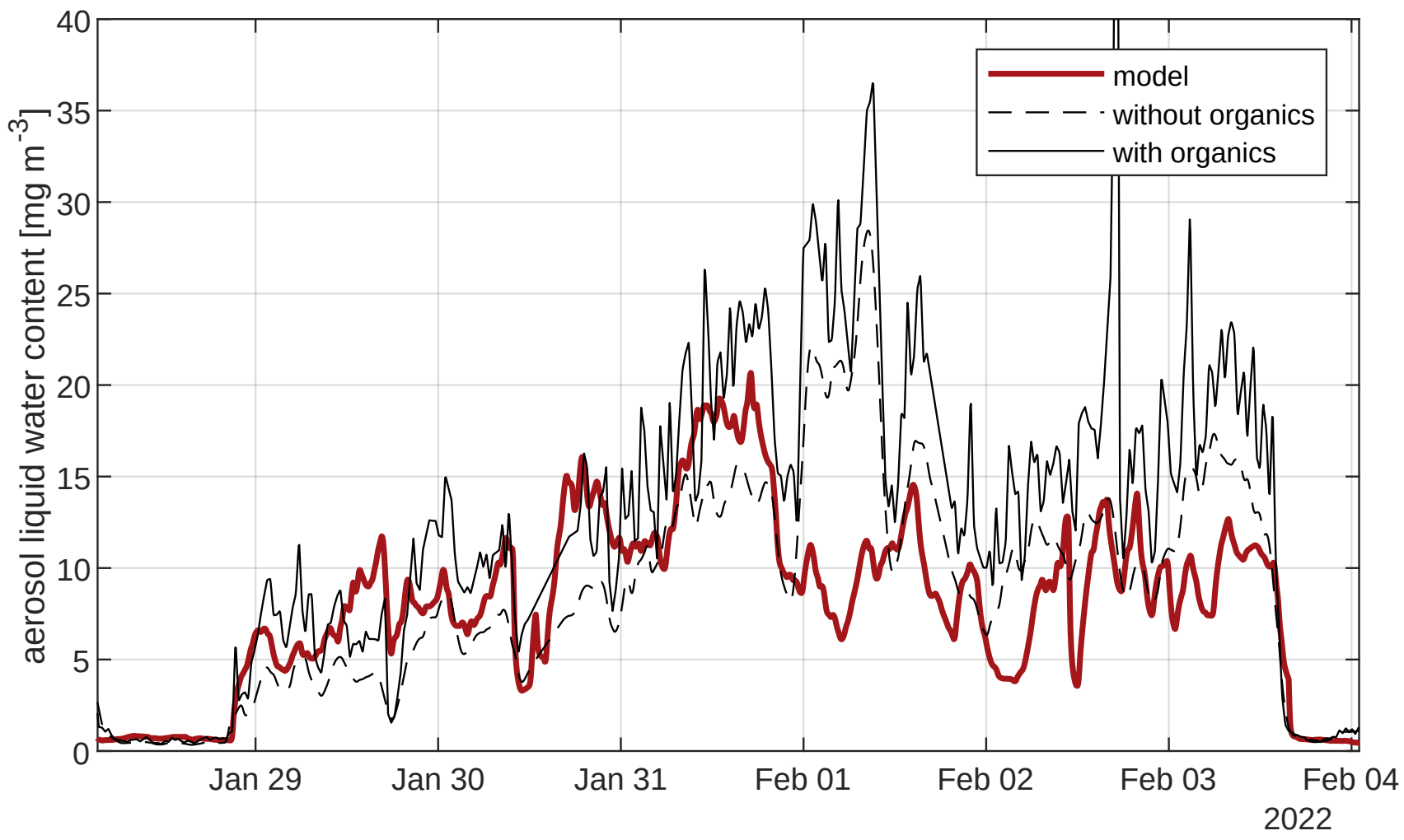

Figure S4: Aerosol liquid water content modeled by [3] based on the assumption of an internally mixed aerosol phase in thermodynamic equilibrium (TE), with (drawn) and without organics (drawn) using two different TE models. The liquid water content in our model runs (red line), which is informed by particle number and radius measurements (see also Fig. S1) agrees well and highlights that our assumptions on the aerosol liquid water content generally agree with TE models. Note that deviations are expected due to uncertainties and different assumptions on the aerosol mixing state.

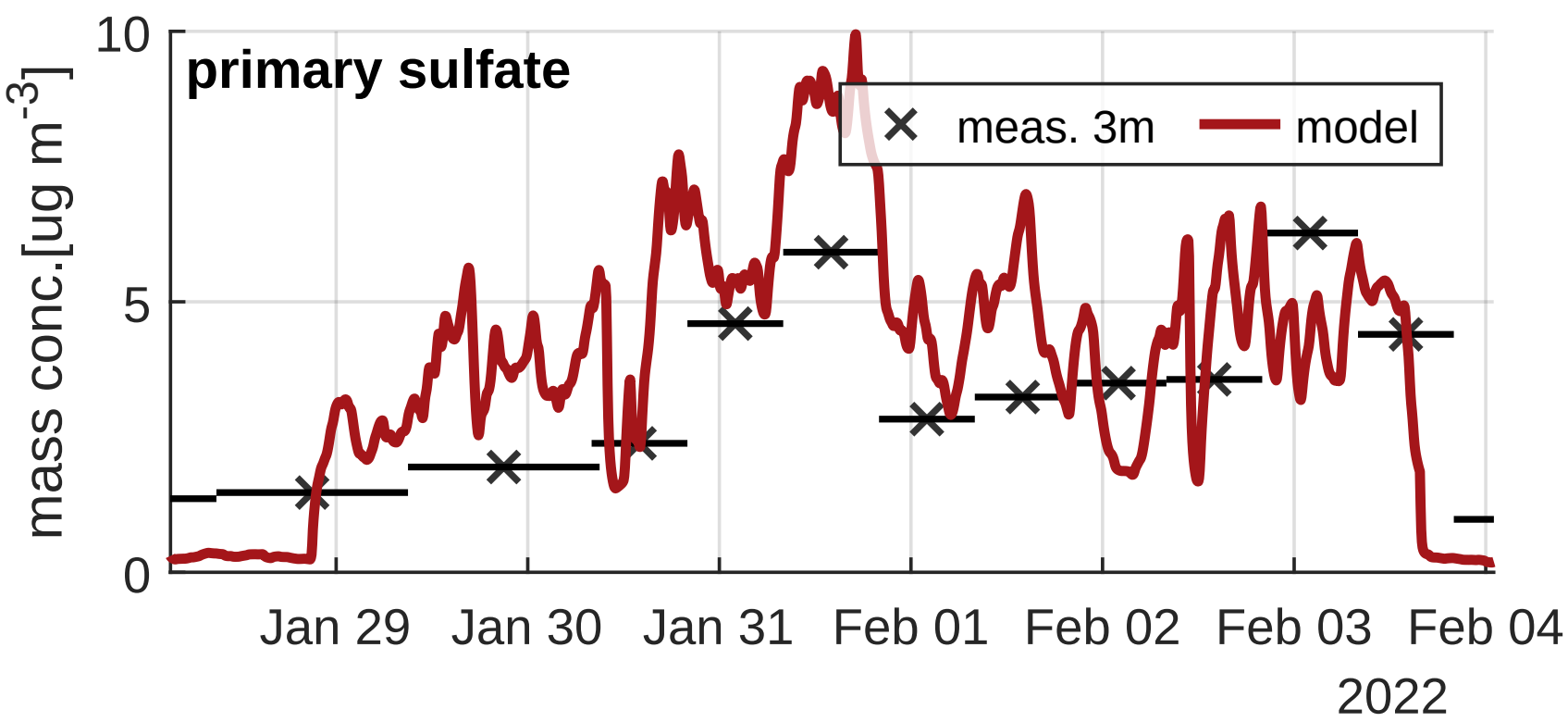

Figure S5: Sulfate mass concentration in PACT-1D due to primary sulfate emissions (red line). Measured primary sulfate (black crosses) by [14].

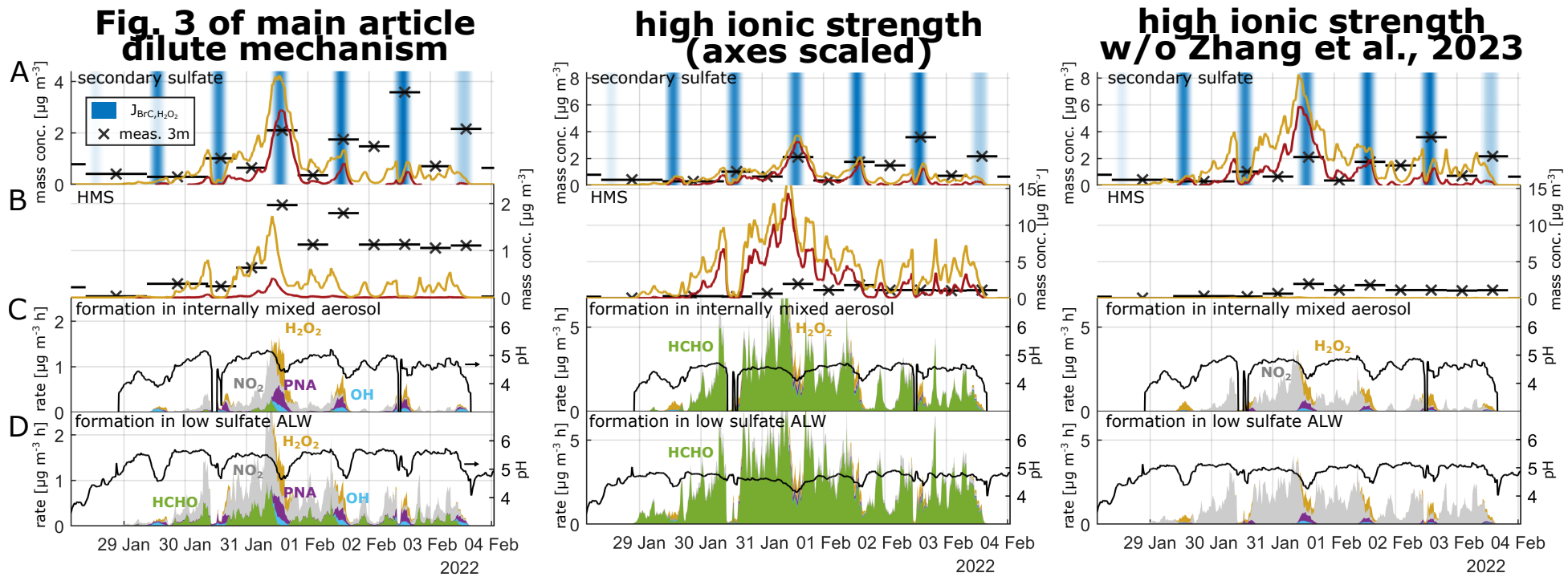

Figure S6: Fig. 3 of the article alongside model results with the aqueous chemical mechanism modified according to recent findings in high ionic strength influences (as indicated in Tab. S5). Increased HMS formation rates in high ionic strength aerosol as found by [27] would cause too high HMS levels (middle panel). The right panel shows a model run with all high ionic strength modifications except that of the HMS formation.

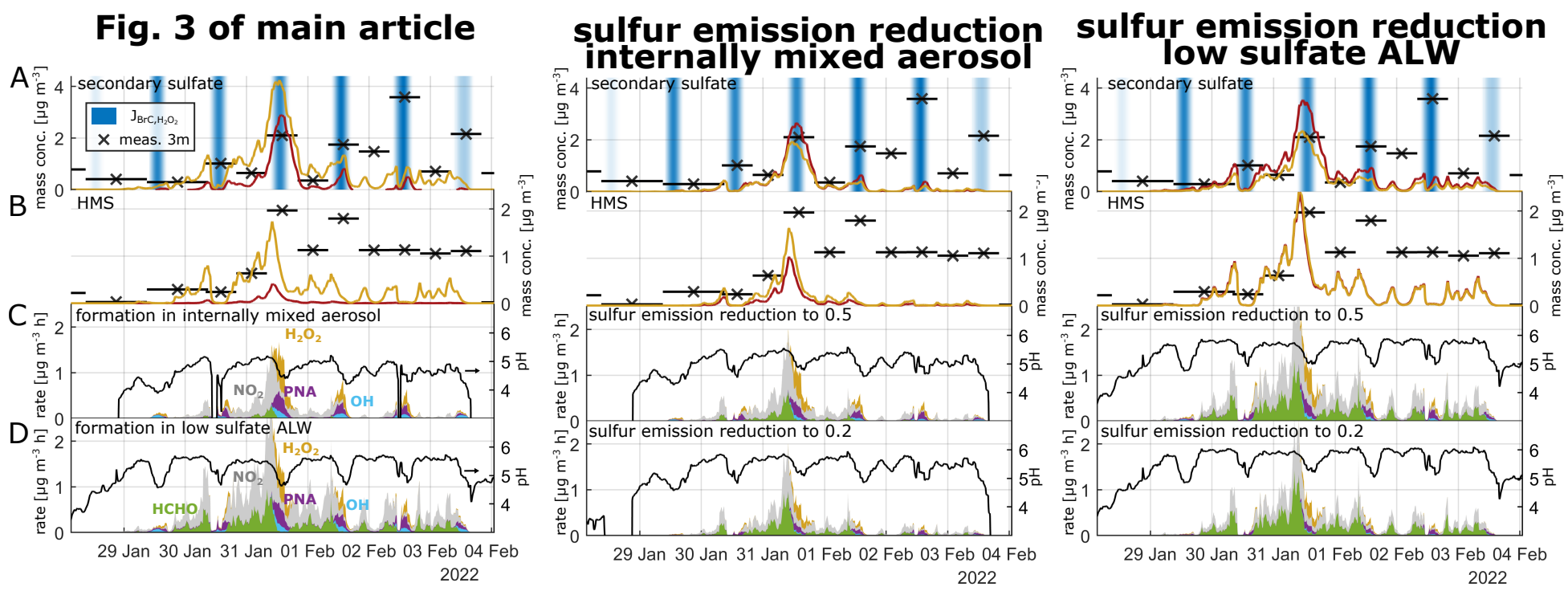

Figure S7: Sulfate and HMS formation after the reduction of sulfur (particulate sulfate and SO_2) emissions in internally mixed aerosol (middle) and the low sulfate ALW component (right panel) of an externally mixed aerosol. The reduction of particulate sulfate emissions cause higher levels of ammonia in the gas phase and thus increases the aerosol pH. The corresponding enhanced rates of HMS formation and sulfate oxidation via NO_2 partly compensate the precursor (SO_2) emission reduction.

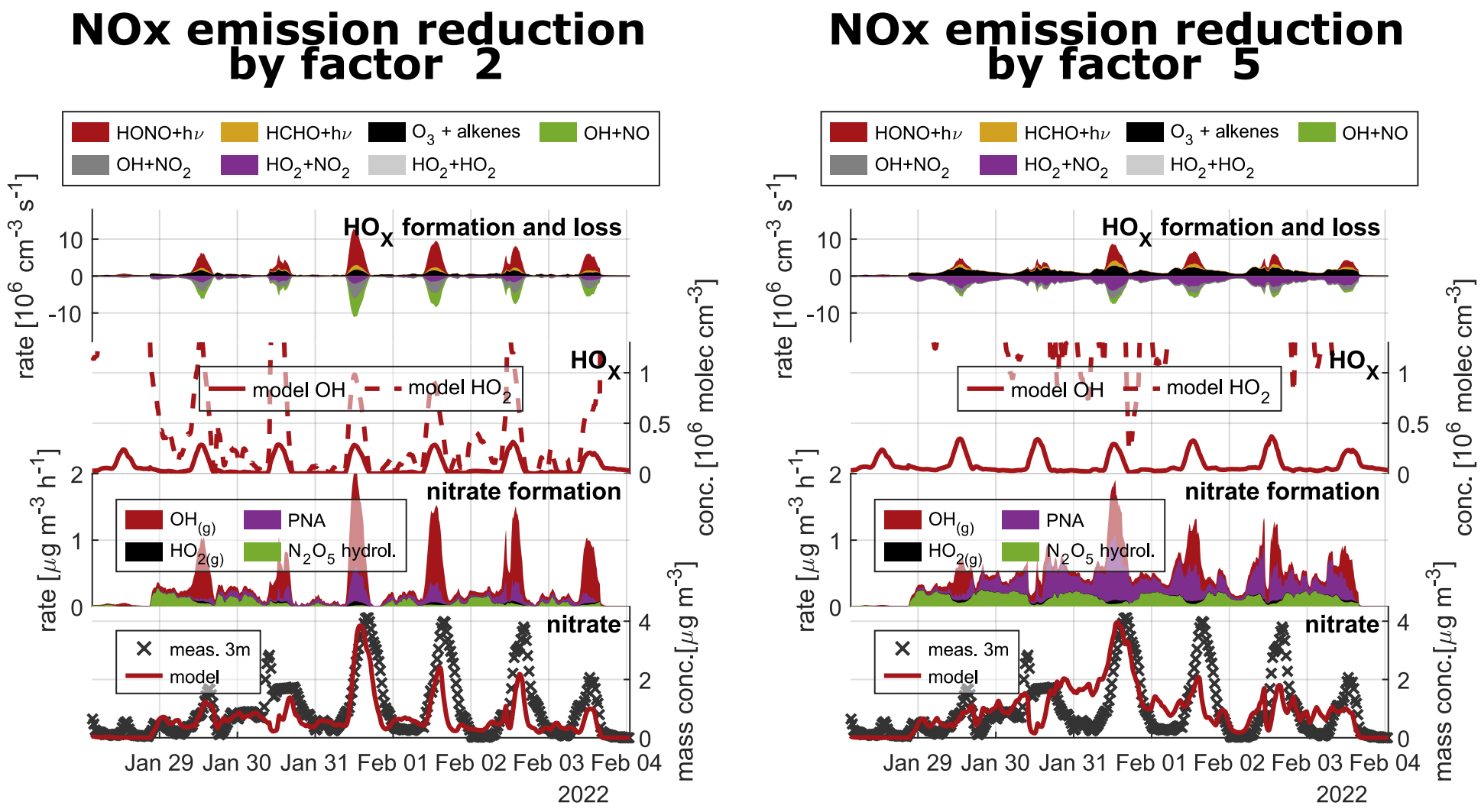

Figure S8: HO_X chemistry and nitrate formation for the model runs with reduced NO_X (and associated HONO) emissions (see Fig. 4 of main article). Nitrate formation during night increases due to N_2O_5 chemistry and PNA formation. Nighttime HO_X is formed predominantly by the reaction of O_3 with alkenes.

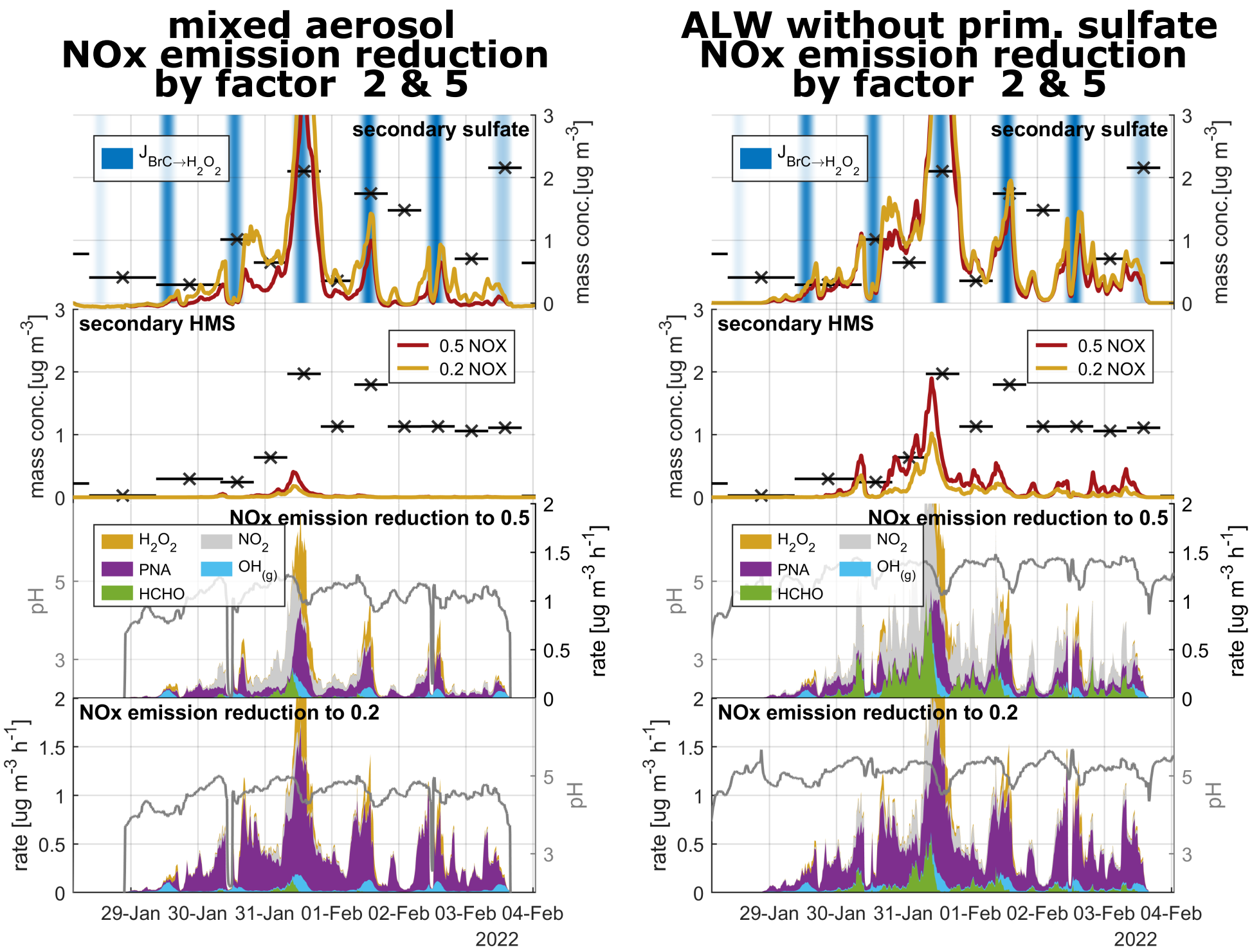

Figure S9: Rates corresponding to the average values shown in Fig. 4E in the article. In both types of aerosol, the reduction of NO_X leads to enhanced production of sulfate. Aerosol pH gets lower through enhanced production, favoring the PNA sulfate oxidation pathway and reducing HMS formation

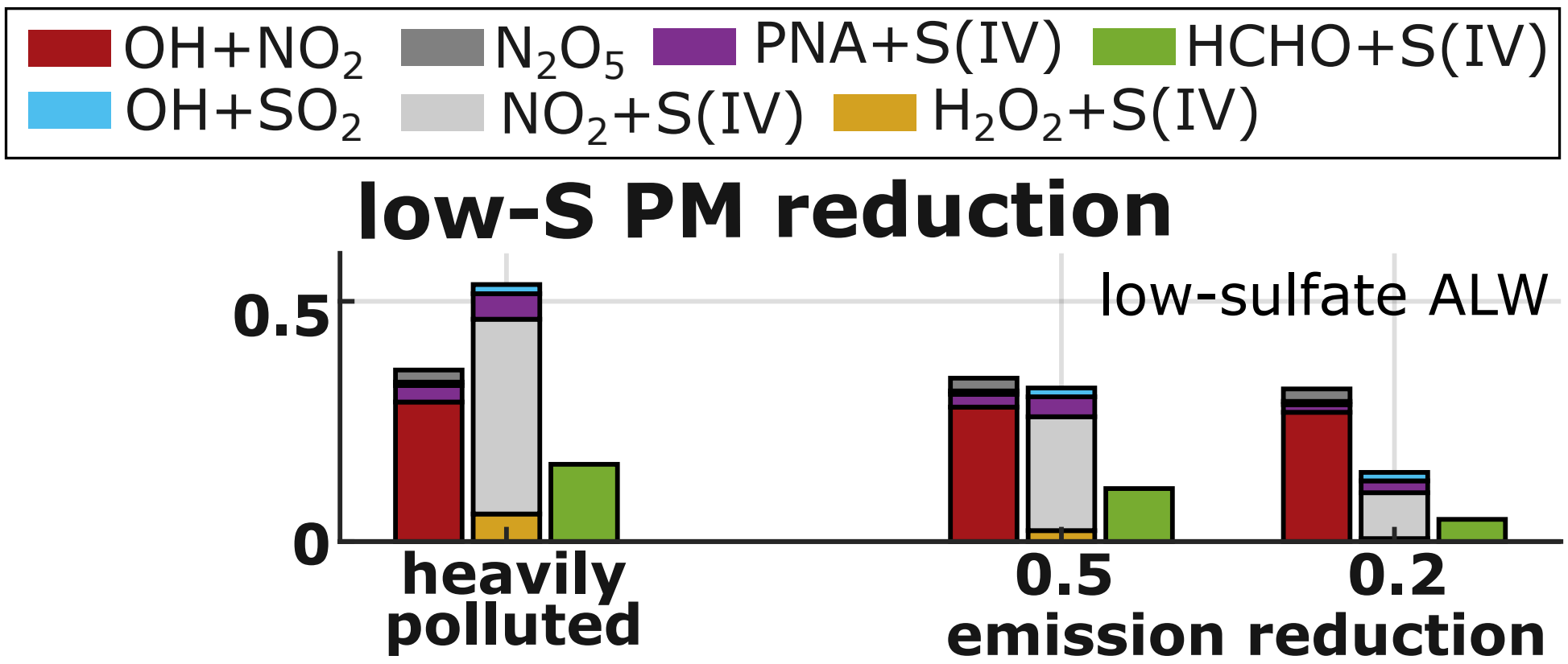

Figure S10: Effect of the reduction of organic PM emissions (e.g. residential wood burning): The low sulfate, higher pH ALW component of the externally mixed aerosol would be reduced, and thus, in-particle formation, particularly the processes that require high pH, would decrease almost linearly.

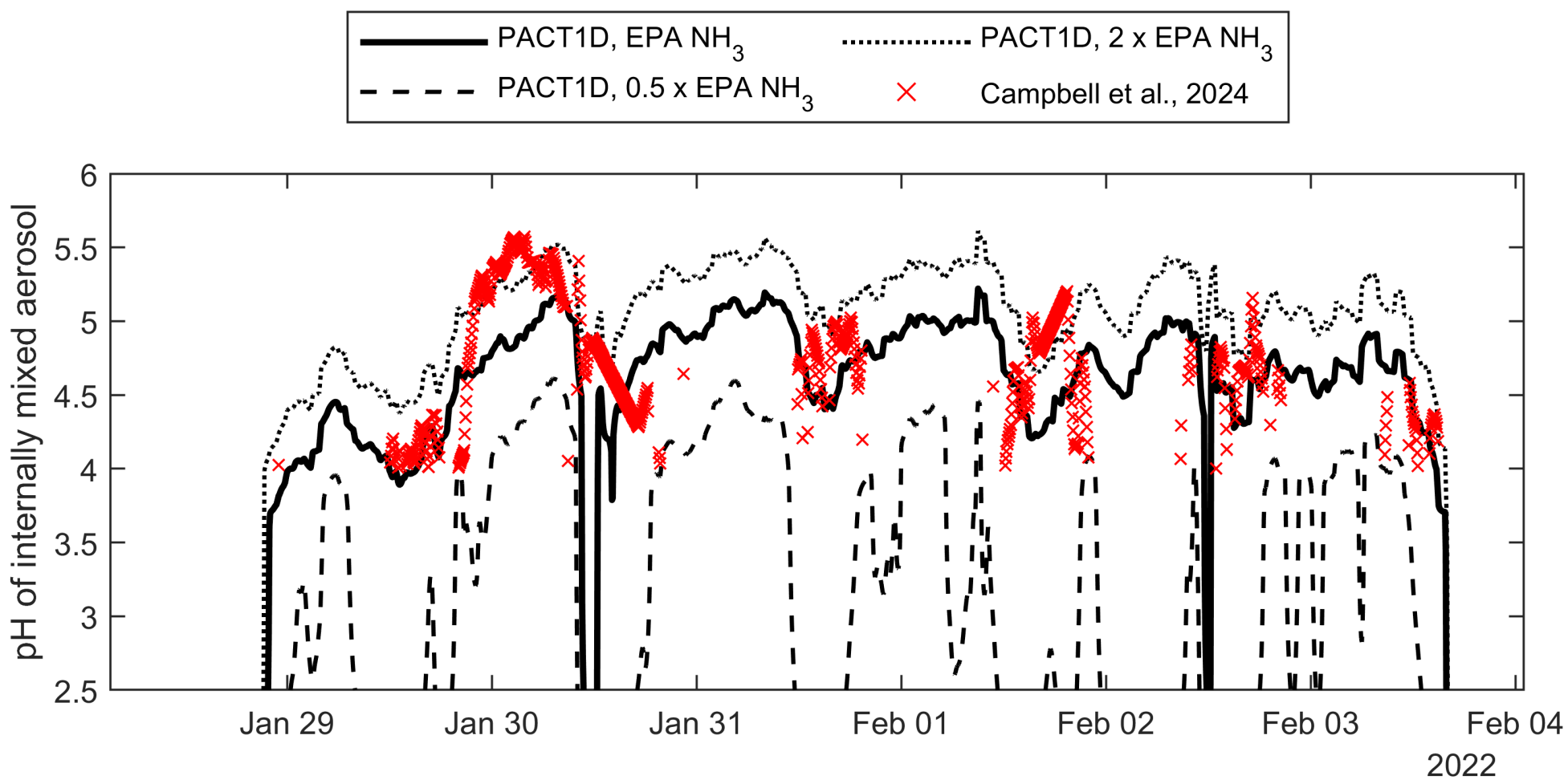

Figure S11: The high mode of the pH of the internally mixed aerosol derived from thermodynamic equilibrium models during the study period by Campbell et al., 2024 ([3], red crosses) agrees well with our kinetically modeled pH for the internally mixed aerosol case when using the EPA-reported ammonia emissions (solid black line). The dashed and dotted lines illustrate the sensitivity of aerosol pH to ammonia emissions, corresponding to halved and doubled emissions, respectively. The good agreement indicates that the EPA-reported ammonia emission rates are accurate.
